# Improved delivery of broadly neutralizing antibodies by nanocapsules suppresses SHIV infection in the CNS of infant rhesus macaques

**DOI:** 10.1371/journal.ppat.1009738

**Published:** 2021-07-20

**Authors:** Jing Wen, Tracy Cheever, Lan Wang, Di Wu, Jason Reed, John Mascola, Xuejun Chen, Cuiping Liu, Amarendra Pegu, Jonah B. Sacha, Yunfeng Lu, Nancy L. Haigwood, Irvin S. Y. Chen

**Affiliations:** 1 Department of Microbiology, Immunology and Molecular Genetics, David Geffen School of Medicine, University of California Los Angeles (UCLA), UCLA AIDS Institute, Los Angeles, California, United States of America; 2 Oregon National Primate Research Center, Oregon Health & Science University, Beaverton, Oregon, United States of America; 3 Department of Chemical and Biomolecular Engineering, School of Engineering, UCLA, Los Angeles, California, United States of America; 4 Vaccine & Gene Therapy Institute, Oregon Health & Science University, Beaverton, Oregon, United States of America; 5 Vaccine Research Center, National Institute of Allergy and Infectious Diseases, NIH, Bethesda Maryland, United States of America; Emory University, UNITED STATES

## Abstract

Broadly neutralizing antibodies (bNAbs) directed to HIV-1 have shown promise at suppressing viremia in animal models. However, the use of bNAbs for the central nervous system (CNS) infection is confounded by poor penetration of the blood brain barrier (BBB). Typically, antibody concentrations in the CNS are extremely low; with levels in cerebrospinal fluid (CSF) only 0.1% of blood concentrations. Using a novel nanotechnology platform, which we term nanocapsules, we show effective transportation of the human bNAb PGT121 across the BBB in infant rhesus macaques upon systemic administration up to 1.6% of plasma concentration. We demonstrate that a single dose of PGT121 encased in nanocapsules when delivered at 48h post-infection delays early acute infection with SHIV_SF162P3_ in infants, with one of four animals demonstrating viral clearance. Importantly, the nanocapsule delivery of PGT121 improves suppression of SHIV infection in the CNS relative to controls.

## Author summary

In patients where HIV-1 is fully suppressed by antiretroviral drugs, HIV-1 still persists in reservoirs. If antiretroviral drugs are stopped, the virus will emerge from these reservoirs and re-seeds systemically. The central nervous system (CNS) is proposed to be a tissue compartment that harbors other HIV-1 reservoirs. A key obstacle that constrains the treatment for the CNS infection is the blood–brain barrier (BBB), a highly restrictive barrier separating the circulating blood from the brain and extracellular fluid in the CNS, which impedes ~98% of the small molecule therapeutics and almost all macromolecules including broadly neutralizing antibodies (bNAbs) directed to HIV-1. Our “nanocapsule” strategy is based on a nanotechnology wherein bNAb molecules are encapsulated within nanocapsules of which the surface contains abundant choline and acetylcholine analogues. This design allows the nanocapsules to effectively cross the BBB to deliver bNAbs into the CNS upon systemic administration and show an impact of bNAb on CNS reservoirs in SHIV infected infant macaques.

## Introduction

Prior to combination anti-retroviral therapy (cART), HIV-1 was shown to infect the central nervous system (CNS) at high levels, causing neurological disorders, the most severe of which was HIV-associated dementia (HAD). Our group was among the first to characterize and isolate HIV-1 from brain tissue of AIDS patients with AIDS-related subacute encephalopathy [1–4]. This along with subsequent studies demonstrating high sequence variation in HIV-1 from brain tissue suggested that there was active and ongoing replication within the CNS [5–12]. HIV infection in the CNS can be detected as early as at 8 days post-infection [13]. In patients whose HIV-1 is fully suppressed by cART, HIV-1 persists in reservoirs. One tissue compartment proposed to harbor HIV-1 is the CNS [14–18]. With the advent of cART, HAD is now quite rare, but other forms of neurological disorders, generally termed HIV-associated neurocognitive disorder (HAND), persist in estimated 15–55% of HIV+ individuals [19,20]. If cART is stopped, virus emerges from these reservoirs and re-seeds systemically. Moreover, 10% of cART-suppressed patients still have detectable viral RNA in the cerebral spinal fluid (CSF) [21]. It has been shown in infected non-human primates (NHPs) that either viral DNA or RNA was detected in the CNS of all non-rebounders when cART was interrupted [18]. Although the exact causes of HAND are unclear, persistent low-level HIV-1, viral proteins, inflammation, or their combinations are expected to lead to neuronal damage [22–29]. Two brain cell types are known to be infected by HIV-1: 1) myeloid lineage cells, including monocytic cells, microglia, and perivascular macrophages; and 2) astrocytes [30–32]. Myeloid lineage cells comprise up to 12%-20% of the total glial cell population in brain, which results in over a billion potential HIV-1 target cells [33]. Therefore, HIV-1 suppression in the CNS is critical.

Passive immunotherapy with broadly neutralizing HIV-1-specific antibodies (bNAbs) is a promising approach for HIV-1 suppression. To perform effectively as a virologic suppressor, antibody levels must be maintained at threshold levels through either transducing the host to maintain endogenous expression [34,35] or multiple dosing [36]. In addition, the use of bNAbs for therapeutic purposes is confounded by emerging viruses that are resistant to single antibodies, strong immunogenicity of idiotypes, and poor penetration into potential reservoir sites such as the CNS. Despite this, bNAb monotherapies directed at HIV-1 have shown promise in suppressing SHIV viremia in primate models [37]. Furthermore, using different combinations of bNAbs has decreased rebound from HIV-1 latent reservoirs in humanized mice [38,39]. To date, over 1,000 infected individuals have received infusion of bNAbs [40]. Available reports on the clinical trials testing 3BNC117, 10–1074, and VR01 show transient reduction of HIV-1 viral loads (VL), delay in viral rebound, and restriction of viral populations emerging from the reservoirs [41–43]. Two clinical trials utilizing the combination of 3BNC117 and 10–1074 showed more effective suppression for extended periods of time than monotherapy [44,45]. However, the available clinical information on bNAbs is limited. Therefore, the preclinical studies in infected macaques can support further testing and deep understanding of bNAbs for further clinic uses in patients. Our recent studies in newborn/infant macaques demonstrated that bNAb cocktails of VRC07-523 and PGT121 can clear viral RNA and DNA in tissues and blood only if administered within 24 or 30 hrs of infection [46,47]. In contrast, the bNAb cocktail treatment beginning at 48 h resulted in durable control of plasma viremia in only half of the treated animals [47]. This age of macaques has been modeled because newborns are exposed to HIV primarily during birth, and intervention with bNAb cocktails in the hours and days following birth could be a tractable approach to prevent acquisition and disease. To date, no published studies have achieved durable clearance in older animals. However, there is evidence of a window of opportunity for influencing the severity of SHIV infection, treatment with VRC07-523 and PGT121 at 10 days post-infection in juveniles limited the seeding of viral reservoirs [48]. A separate study showed that bNAb cocktail treatment in older animals at 3 days post-infection resulted in significant attenuation of the infection, concomitant with CD8+ T cell responses [49]. Therefore, the insufficient clearance is associated with latent and/or persistently replicating HIV-1 hides in more established reservoirs in sanctuary sites, such as the CNS, where antibody penetration is low [50]. Although bNAb therapy is effective for treatment of systemic infection, crossing the BBB is a major obstacle to apply bNAbs for CNS infection. Systemic administration of monoclonal antibodies (mAbs) is ineffective for treatment of metastatic CNS cancers [51–60]. Similarly, access of potential HIV-1 reservoirs in the CNS is not possible with current approaches. Nanotechnology has shown promise for CNS delivery of macromolecules [61,62], but effective CNS delivery via the intravenous route is still challenging.

To address these limitations of bNAb therapy on HIV-1 infection in the CNS, we used a nanocapsule platform developed by our group, by which individual macromolecules are encapsulated within a thin polymer shell. By encapsulating macromolecules within zwitterionic nanocapsules containing abundant choline and acetylcholine analogues, encapsulated macromolecules can be effectively delivered to the CNS with >10-fold higher levels than native forms upon systemic administration [63–68]. We recently published effective delivery of rituximab (RTX, anti-CD20) and nimotuzumab (Nimo, anti-EGFR) by nanocapsules through intravenous route to treat primary and metastatic brain tumors [67,68]. In a xenografted CNS metastatic Non-Hodgkin Lymphoma (NHL) model in humanized mice, native RTX is ineffective, while treatment by nanocapsulated RTX demonstrated elimination of the tumor burden, including in the brains [68]. Therefore, we have demonstrated the capability of nanocapsules to deliver mAbs crossing the BBB and provide a means to treat CNS diseases with bNAb immunotherapy.

NHPs infected by chimeric simian-human immunodeficiency virus (SHIV) are arguably one of the best animal models for HIV-1 infection of humans for the study of bNAbs. Infant NHPs with SHIV infection exhibit high viral loads and rapid virus dissemination, reaching distal tissues within one day of exposure, which allows in-depth investigations of viral dissemination and effectiveness of bNAb treatments in a very stringent model [69]. Our group has demonstrated that bNAbs are highly effective at systemic suppression and are capable of clearing SHIV in infected NHP infants [47]. However, studies on the therapeutic impact of bNAbs in infants’ CNS are not available. We herein test the therapeutic effect of encapsulated bNAbs in SHIV-infected infant NHPs at 48 hr post-infection, simulating acute infection, and at Week (WK) 11 post-infection, which was considered as chronic infection. Overall, both the maintenance and the CNS delivery of the bNAb PGT121 was significantly improved by nanocapsules compare to the native form. A single dose of nanocapsules injected at 48 hours showed suppression on viral load in plasma comparable to four doses of native cocktail treatment as in our published study [47]. Importantly, nanocapsulated bNAbs have a more pronounced effect on CSF VL in SHIV-infected NHPs. Taken together, the nanocapsules can enable effective delivery of bNAbs into the CNS, opening a new avenue for treatments of HIV-1 CNS infection.

## Results

### Understanding the nature of CNS infection in infant SHIV-infected macaques

**Fig 1:**
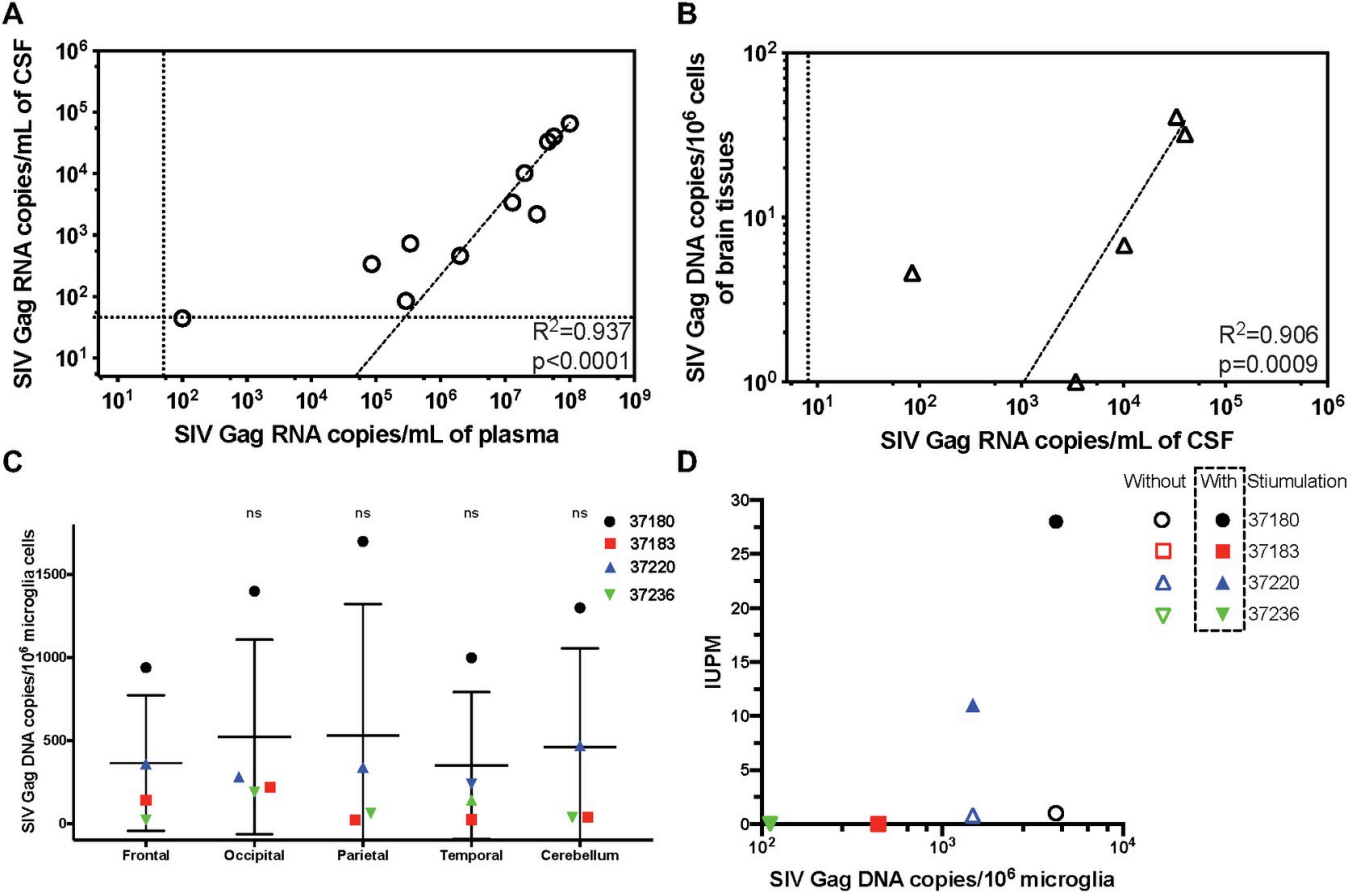
Infection occurs in the central nervous system (CNS) of infant rhesus macaques. 11 one-month-old infant rhesus macaques were exposed to a single high dose SHIV_SF162P3_ challenge by atraumatic oral inoculation. A) Correlation between SHIV_SF162P3_ Gag RNA copies in plasma and CSF in infected infant rhesus macaques. The animals showing positive RNA copies in both plasma and CSF are included in the figure. Dotted lines indicate limits of detection. Each dot represents one animal, and all data are fitted by nonlinear log-log line regression. R^2^ and p value indicate the fitness of Pearson correlation. Dotted lines indicate limits of detection. B) Correlation between SHIV_SF162P3_ Gag RNA copies in CSF and vDNA copies in brain tissue. Animals showing positive RNA copies in both CSF and vDNA are included in the figure. Each dot represents one animal, and all data are fitted by nonlinear log-log line regression. R^2^ and p value indicate the fitness of Pearson correlation. Dotted lines indicate limits of detection. C) Comparable viral DNA copies in microglia cells in five brain locations. The mean of vDNA copies in microglia from five brain locations are compatible. Data are shown as means ± s.d. of four animals. Statistical significance, compared with the vDNA in microglia from Frontal, was determined by two-tailed unpaired t test. ns: not significant. D) Inducible virus is correlated with vDNA in microglia. The inducible virus was detected viaTZA assay. IUPM, infectious units per million microglia cells. Primary microglia were stimulated with 20 ng/mL M-CSF and 25 ng/mL IL-10 for 5 days. Both Without and With Stimulation fit Pearson correlation. Without Stimulation: R^2^ = 0.771, p = 0.1219; With Stimulation: R^2^ = 0.9854, p = 0.0073.

The *in vivo* CNS delivery of therapeutics may be determined by sampling brain tissue, cerebrospinal fluid (CSF), or extracellular of interstitial fluid (ISF) after systemic administration [70,71]. Although brain tissues provide direct evidence for CNS delivery, their sampling is costly and impossible to facilitate longitudinal analysis. The measurement of drug concentrations in brain ISF is also challenging due to the anatomical confinement of the organ and the separation of ISF and blood by the BBB, which requires microdialysis. In contrast, CSF is considered as the most practical approach in terms of effort, cost and throughput. Serial CSF sampling can be performed with relatively little risk, which affords detailed information on therapeutic concentration time course in the CNS. Moreover, the analysis of CSF remains among the most important procedures for diagnosing CNS infection [72,73]. Therefore, we use CSF samples as a surrogate measure of both SHIV infection and CNS delivery of bNAbs in this study. To attain a better understanding of the nature of SHIV infection in the CNS, we exposed 11 infant NHPs (24–28 days old) via atraumatic oral inoculation (swallowing) with a dose of 2 mL (4.1×10^4^ TCID_50_) of cell-free SHIV_SF162P3_ stock. The Envelope of this SHIV was cloned from the CSF of an HIV-infected subject [74]. We were performing other infection studies in infants at the time and could confirm contemporaneous infection of untreated control animals. All NHPs were monitored longitudinally for the VL in their plasma and CSF as well as viral DNA (vDNA) in brain tissues at necropsy (**S1 Table**). In our previous study, untreated infant NHPs infected by the same in vivo titered stock of SHIV_SF162P3_, whose parent strain HIV_SF162_ was originally derived from the brain of an AIDS patient, showed disseminating viral infection in all tissues by days 7–14 post-infection resulting in sustained postacute plasma VL >10^7^ copies/ml [46]. We detected both plasma and CSF VL in all 11 animals as well as vDNA in brain tissues in 5 out 11 animals at the time of necropsy. Viral RNA was detected in CSF with a strong correlation with RNA copies in plasma (*R*^2^ = 0.949) (**Fig 1A**). Plasma VL averaged ~ 3 logs higher than CSF VL. Furthermore, the vDNA copies detected in brain tissues of the 5 aforementioned animals are correlated with the amount of VL in CSF (*R*^2^ = 0.906), indicating that infection in these two compartments of the CNS is related and connected (**Fig 1B**). The correlation is also observed between vDNA in brain and VL in plasma (*R*^2^ = 0.881) (**S1 Fig**).

One of the cell types in the brain infected by SHIV is brain resident myeloid cells known as microglia [75,76]. By purifying microglia from brain tissue, we expect to concentrate infected cells to support an accurate detection of vDNA in brain tissue. To understand the SHIV distribution in brain, microglia were isolated from four lobes of the cerebrum, as the frontal lobe, occipital lobe, parietal lobe, and temporal lobe, as well as cerebellum by Percoll gradient sedimentation. After concentration, the microglia and macrophage percentages increased by up to 65%-90%, which was confirmed by CD45+CD11b+ expression (**S2 Fig**). The vDNA copies in microglia from different regions were comparable, indicating non-preferential distribution of infection in brain (**Fig 1C**). To further confirm the inducibility of latent virus in microglia, the TZA assay, a TZMbl-based quantitative viral outgrowth assay, was used to quantify replication-competent virus levels. Since infection distributed evenly in the brain, microglia isolated from different brain regions of the same animal were mixed and stimulated by M-CSF and IL-10. Animals harboring more than 10^3^ vDNA copies per 10^6^ microglia showed replication-competent virus correlated with vDNA copies (**Fig 1D**). These results indicate that CNS infection, showing detectable VL in CSF and vDNA in brain tissues, is correlated with plasma VL. Moreover, the vDNA detected in microglia is capable of producing replication-competent virus in the CNS with stimulation.

### bNAb-based treatments show significant plasma suppression but limited impact on CNS infection

**Fig 2:**
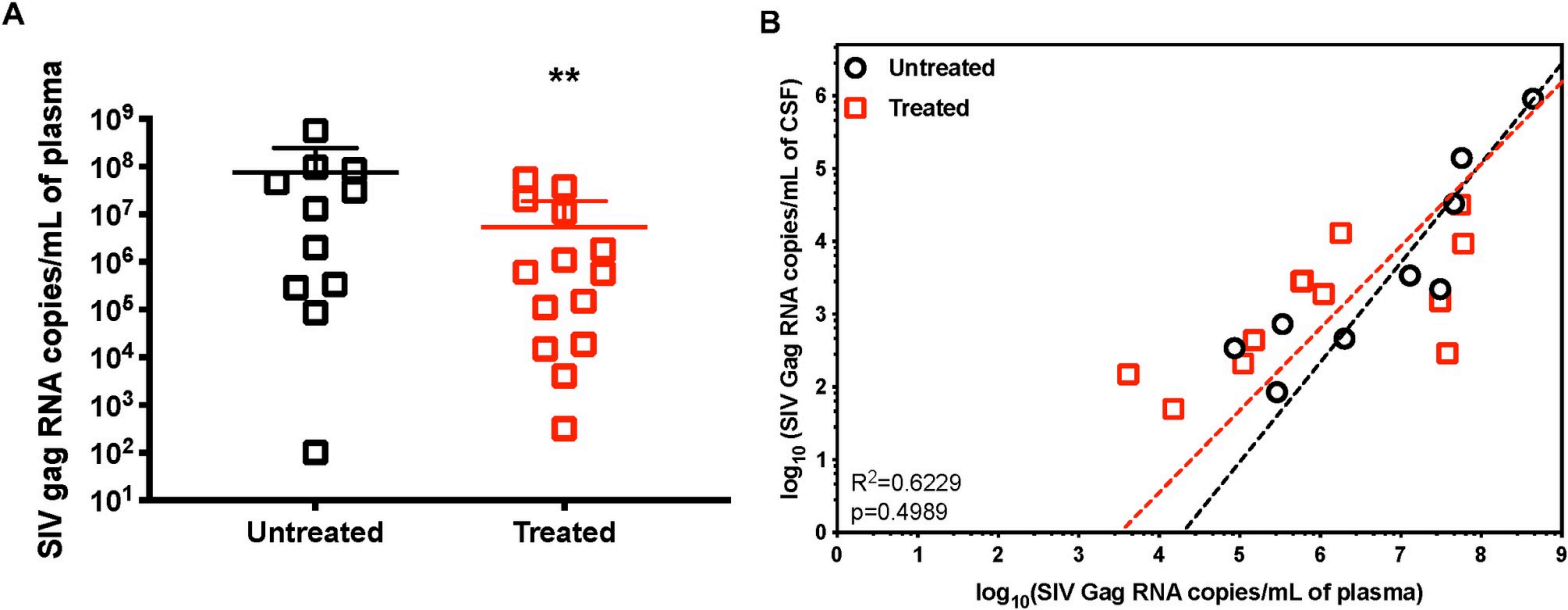
The bNAb-based treatment impacts infection in blood but not in the CNS in infant rhesus macaques. 14 one-month-old infant rhesus macaques were exposed to a single high dose SHIV_SF162P3_ challenge by atraumatic oral inoculation and treated with bNAbs or cART+bNAbs with various doses and regimens at 48h post-infection (details in S1 Table). A) Viral RNA copies in plasma of infant rhesus macaques without treatment (Untreated) and treated with bNAbs or bNAbs+cART (Treated) at 48 h. Statistical significance was calculated to untreated group by two-tailed unpaired t test. **: P values < 0.0021. B) Comparison of correlations between viral RNA copies in plasma and CSF of Untreated and Treatment animals. Each dot represents one animal, and all data are fitted by nonlinear log-log line regression. Significance between the correlations of two groups is calculated by multiple regression model. There is no statistically significant difference between slopes (p-value = 0.4989).

In our previous studies on SHIV-infected infant NHPs [47], we tested the efficacy of bNAb-based treatments, including single and cocktail bNAb treatments, with and without cART **(S2 Table)**. Compared to infected animals without treatment, animals treated with bNAb showed significant decreases in plasma VL (P < 0.0021) (**Fig 2A**). To further understand the impact of bNAb-based treatment on CNS infection, we evaluated the correlation between plasma VL and CSF VL in both treated and untreated animals, whose CSF VL and plasma VL were both detectable. As shown in **Fig 2B**, the plasma-CSF VL correlation is comparable between untreated and treated animals, indicating the impact of bNAbs on CNS infection mainly depends on reducing plasma VL. As discussed in our published study, maximizing the effective concentration and persistence of bNAbs is critical for reducing viral spread [47].

### A polymer-based nanocapsule delivery platform improves bNAb delivery to the CNS

**Fig 3:**
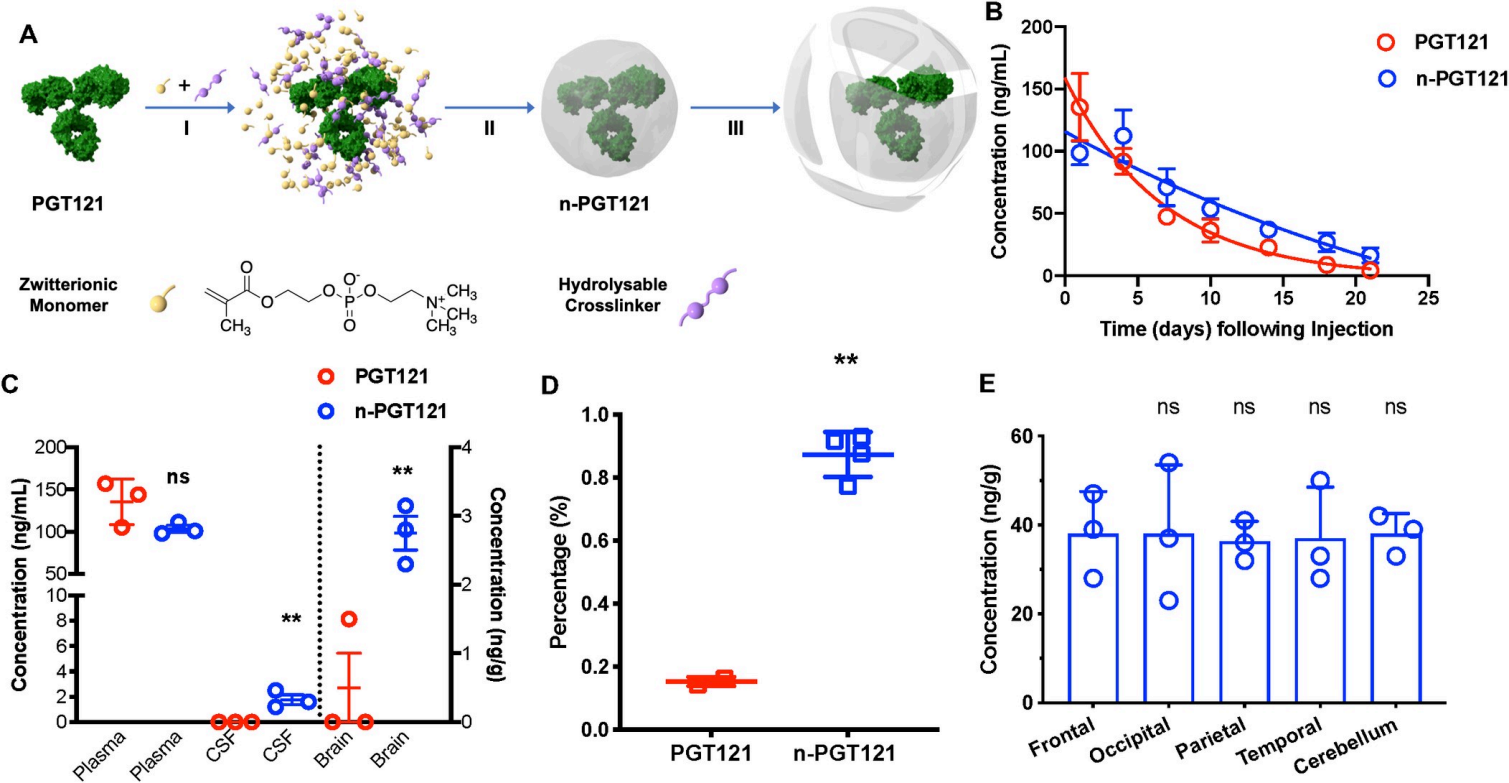
Nanocapsules improve bNAb penetration in CNS. A) Scheme of the synthesis and release of PGT121 nanocapsules (n-PGT121) by (I) enriching zwitterionic monomer (MPC) and hydrolysable crosslinkers (PLA-PEG-PLA) around a PGT121 molecule, (II) in-situ polymerization of the monomer and crosslinkers forming a thin shell of polymer around PGT121, and (III) releasing PGT121 when polymer shells are degraded under physiological condition by hydrolysis of the crosslinkers. B) Kinetics of native PGT121 and n-PGT121 in mice. C57BL/6 mice were randomly separated into two groups and injected with 5 mg/kg of PGT121 (n = 6) and n-PGT121 (n = 6) via retro-orbital injection. The release of PGT121 was assayed by ELISA using ST09 and anti-human IgG. C) Nanocapsules improved CNS delivery (CSF and brain tissue) of PGT121 in mice. C57BL/6 mice were randomly separated into two groups and injected with 5 mg/kg of PGT121 (n = 6) and n-PGT121 (n = 6) via retro-orbital injection. Body fluids and tissues were collected 3 days after injection. The concentrations of native and released PGT121 in plasma, CSF, and perfused brain homogenates were measured by ELISA. Data are shown as means ± s.d. of three animals. Statistical significance was calculated to the PGT121 group by two-tailed unpaired t test. ns: not significant; **: P values < 0.0021. D) SHIV infected infant rhesus macaques were injected with 5 mg/kg PGT121. The percentage of PGT121 concentration in CSF of that in plasma of infant rhesus macaques. The percentages of PGT121 concentration in CSF and that in plasma from infant rhesus macaques treated with native PGT121 (n = 2) and n-PGT121 (n = 4) on Day 7 after injection. The concentrations of native and released PGT121 in CSF were measured by ELISA. Data are shown as means ± s.d. of each group. Statistical significance was calculated to PGT121 group by two-tailed unpaired t test. **: P values< 0.0021. E) Nanocapsules show a uniform distribution in different brain locations of rhesus macaques. Five brain tissues from n-PGT121 treated SHIV infected infant rhesus macaques were weighted, washed, and homogenized. Supernatant was collected after centrifuging the homogenate. The concentration of released PGT121 was detected by ELISA. Statistical significance was calculated to Frontal by two-tailed unpaired t test. ns: not significant.

Our group developed an antibody nanocapsule platform to achieve over 10-fold higher levels of antibody delivery to the CSF at levels maintained for a significantly longer time than the native form. The formulation procedure of nanocapsules is described in **Fig 3A**. In brief, zwitterionic monomers are enriched around the surface of a bNAb molecule (PGT121 in this study), followed by in-situ polymerization to encase the bNAb molecule to form nanocapsules. Crosslinkers fortify the structure of the polymer coat and provide properties for release of the encapsulated PGT121. We have designed crosslinkers that are hydrolysable under physiologic conditions in the blood and empirically determined ratios of hydrolysable and non-hydrolysable crosslinkers which enable more gradual release of PGT121 over time. The kinetics of nanocapsule release was compared among nanocapsules with 0% hydrolysable crosslinkers (denoted as n-PGT121_intact_), 50% hydrolysable crosslinkers (denoted as n-PGT121), and 100% hydrolysable crosslinkers (denoted as n-PGT121_hydrolysable_) *in vitro* by ELISA. The release only occurs in n-PGT121 and n-PGT121_hydrolysable_, whose half-lives are three days and less than one day, respectively (**S3A Fig**). Furthermore, stability of the nanocapsules was demonstrated with n-PGT121_intact_, which maintained encapsulated structures for at least two weeks. The brain delivery efficiency of n-PGT121_intact_, n-PGT121, and n-PGT121_hydrolysable_ was compared in rats injected with 10 mg/kg via tail vain. Among all samples, n-PGT121 with 50% hydrolysable crosslinkers achieves the highest PGT121 concentrations in both CSF and brain tissues (**S3B Fig**). Based on this pilot study, n-PGT121 was selected for successive experiments. The n-PGT121 exhibit a mean size of ~25 nm, which is significantly different from its native counterpart (~4.5 nm) (**S3C Fig**). **S3D Fig** shows representative transmission electron microscopy (TEM) images of n-PGT121 exhibiting a uniform spherical morphology with a similar average diameter of 30 nm. Our previous work has demonstrated that each core-shell nanocapsule encapsulates one single antibody molecule [68]. The half-life of antibody *in vivo* is determined by multiple factors besides the release kinetics, including absorption to cells and tissues, excretion, and degradation. The *in vivo* kinetics and biodistribution of both native PGT121 and n-PGT121 were further studied in mice with more animal numbers. As shown in **Fig 3B**, PGT121 shows decay kinetics in plasma with a half-life of seven days, which is comparable with the findings in previous publications [77]. In contrast, mice injected intravenously with n-PGT121 showed significantly prolonged retention in the serum, with a half-life of 21 days. For further testing, the improved CNS delivery of n-PGT121 was compared to native PGT121 at Day 3 post-injection. The concentration of free PGT121 released from n-PGT121 was comparable to that of native in plasma. In contrast, twelve- and five-fold enhancements of PGT121 levels were observed in the CSF and brain tissue, respectively, when delivered by n-PGT121 relative to native PGT121 (**Fig 3C**). To evaluate the potential impact of n-PGT121 on the BBB, the BBB integrity of mice treated with native PGT121 and n-PGT121 was tested by commonly-used Evans blue (EB) dye extravasation (**S3E Fig**). EB is not naturally able to pass through the BBB, therefore, its presence in brain tissues indicates alterations in permeability [78]. Compared to the mice with damaged BBB due to the brain tumor growth, both mice treated native PGT121 and n-PGT121 showed comparable EB fluorescence signals in their brain tissues as PBS-treated animals, indicating no impact on BBB integrity by treatments. These results show the prolonged maintenance in plasma, improved penetration into the CNS, and lack of adverse BBB damage associated with treating rodents with n-PGT121.

The enhanced delivery of n-PGT121 into the CNS was further confirmed in SHIV-infected infant NHPs. Infant NHPs were infected orally with SHIV and injected with 5mg/kg of PGT121 (n = 2) or n-PGT121 (n = 4) at 24 hours or 48 hours post exposure, respectively. In accordance with other antibodies showing ~0.1% plasma concentration delivered into the CNS, 0.13% plasma concentration of native PGT121 was detected in the CSF at Day 7 post-injection (**Figs 3D and S4**). Compared to PGT121, n-PGT121-treated NHPs showed CSF concentration of approximately 1.6% plasma concentration, which was 15-fold increase over the native form. To further study whether n-PGT121 penetrates the BBB with any location preference, the concentration of released PGT121 from n-PGT121 was compared among four lobes of the cerebrum, specifically the frontal lobe, occipital lobe, parietal lobe, and temporal lobe, as well as cerebellum at Week 15 post-injection. As shown in **Fig 3E**, all brain regions show similar PGT121 concentration, indicating uniform biodistribution of n-PGT121 in the brain. Compared to native PGT121, this efficient delivery of n-PGT121 in the CNS provides longer antibody persistence at higher concentration with uniform distribution in the brain, which may also be a critical factor for the suppression of the CNS infection.

### Nanocapsules maintain bNAb circulation and deliver bNAbs into the CNS in infant SHIV-infected macaques

**Fig 4:**
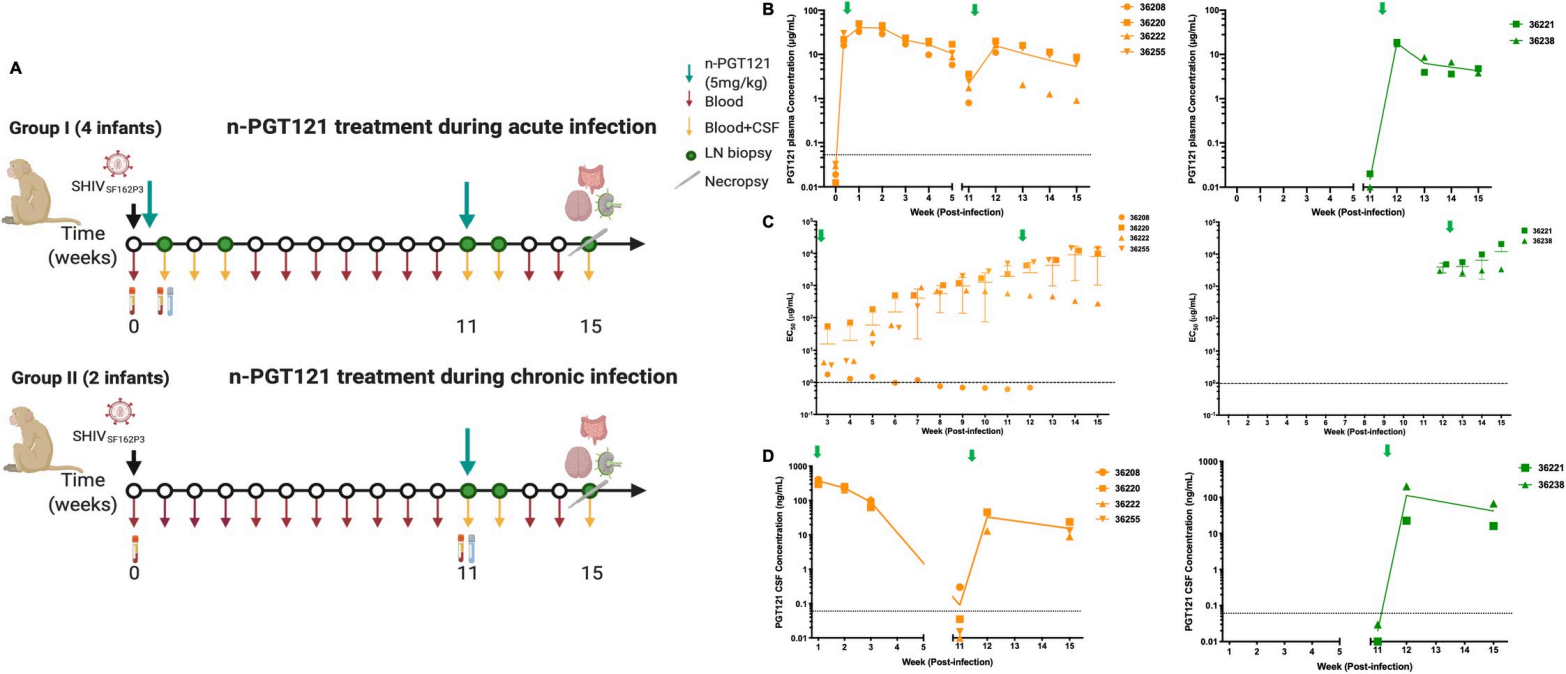
Nanocapsules maintain bNAb circulation and improve penetration in CNS in SHIV-infected infant rhesus macaques. A) Experimental design of the n-PGT121 treatment in SHIV infected infant rhesus macaques at 48 h during acute infection (Group I) and Week 11 (WK11) during chronic infection (Group II). One-month-old infant rhesus macaques negative for Mamu-B*08 and Mamu-B*17 alleles were randomly assigned to groups. All animals were exposed to a single high dose SHIV_SF162P3_ challenge by atraumatic oral inoculation. Black arrows indicate SHIV infection. Green arrows indicate n-PGT121 treatments. Dark red arrows indicate blood collection. Dark yellow arrows indicate CSF collection. Green circles indicate LN biopsies. Scalpels indicate time of necropsy. B) The released PGT121 concentration from n-PGT121 in plasma in acute infection group (Group I, n = 4) and chronic infection group (Group II, n = 2). WK0 and WK11 samples were collected before n-PGT121 infusion. Data are shown as means ± s.d. of each group. Green arrows indicate n-PGT121 treatments. C) Seroconversion was determined by measuring the concentration (EC_50_) of envelope antibodies in the plasma of the acute infection group (Group I, n = 4) and chronic infection group (Group II, n = 2). EC_50_ was assayed by an SHIV_SF162_ gp140-specific ELISA. Data are shown as individual data for each animal and means ± s.d. of each group. Pre-treatment plasma (day 0) was used as a negative control for the assay. WK11 samples for this assay were collected before n-PGT121 infusion. Green arrows indicate n-PGT121 treatments. D) The released PGT121 concentration from n-PGT121 in CSF in acute infection group (Group I, n = 4) and chronic infection group (Group II, n = 2). WK0 and WK11 samples were collected before n-PGT121 infusion. Data are shown as means ± s.d. of each group. Green arrows indicate n-PGT121 treatments.

To evaluate of the therapeutic effect of n-PGT121, the impact on viral suppression was studied in infant NHPs infected with SHIV by the oral route. The study design is schemed in **Fig 4A**. We inoculated 6 one-month-old rhesus macaques orally with SHIV_SF162P3_ on Day 0 and intravenously treated them with n-PGT121 (5mg/kg) at both Day 2 and Week 11 (Group I, n = 4), which mimics early treatment during acute infection, or only at Week 11 (Group II, n = 2), which represents delayed therapy during chronic infection, and monitored antibody kinetics and virologic outcomes in both blood and the CNS for up to 15 weeks. Blood samples were collected weekly, while CSF samples were obtained at Week 1, 2, 3, 11, and 12 post-infection in Group I and only at Week 11 and 12 in Group II. Additionally, lymph node (LN) biopsies were performed at Week 1, 3, 11 and 12 in Group I and at Week 11 and 12 in Group II. Necropsy was completed at Week 15 post-infection for both groups.

We evaluated the kinetics of free PGT121 released from n-PGT121 in plasma by ELISA assays in Group I animals (**Fig 4B**). Peaks of PGT121 plasma concentrations occurred at Week 1 post-injection, indicating a gradual release of n-PGT121; however, the decay kinetics were different between two injections. As fitted with the one-phase decay model, n-PGT121 showed a > 6-week half-life of PGT121 from first the injection on Day 2 and a 2.5-week half-life of the repeat treatment at Week 11, which were both longer than the 15.9-day half-life of native PGT121 with 4 doses [47]. To assess the seroconversion *in vivo* plasma samples from infected infant macaques were tested for binding to HIV envelope gp140 by ELISA. Antibody concentration was quantified by calculating the envelope antibodies in plasma (EC_50_) at different time points (**Fig 4C**). The EC_50_ of the first injection in Group I was 14.6 ng/mL, which is comparable to that obtained from purified PGT121 specific to the same SHIV_SF162P3_ virus stock (12.8 ng/mL) [46]. After antibody decay, there was seroconversion in 3 of the 4 treated infants and no detectable antibody produced in the animal with undetectable viremia (Animal 36208). Animal 36222 had a lower level of seroconversion that was declining by Weeks 11–15, which is common in babies with very high viremia. To confirm the brain delivery of n-PGT121, the CSF concentration of released PGT121 from n-PGT121 was also tested with the ELISA assay (**Fig 4D**). All animals in Group I showed consistent CSF delivery efficiency: PGT121 in the CSF was ~370 ng/mL at Week 1, approximately 1.6% of plasma levels, then dropped down to 90 ng/mL at Week 3. The brain delivery efficiency of n-PGT121 at Week 1 was 15-fold higher than that of native PGT121, with only 0.13% of plasma levels. Before the second injection at Week 11, the CSF concentration was under detectable levels. The second injection of n-PGT121 increased the CSF concentration up to 35 ng/mL, corresponding to 0.3% of its plasma level. These findings indicate that n-PGT121 shows prolonged half-life and enhanced CNS delivery in infant NHPs with acute infection.

In contrast, the half-life of n-PGT121 in Group II animals injected at Week 11 was about one week, despite it being the first injection (**Fig 4B**). The EC50 of envelope antibodies was above 3000 ng/mL, indicating the animals were seropositive at the time of PGT121 infusion at Week 11 (**Fig 4C**). The two animals showed CSF concentrations with wide variation at Week 12, with the higher concentration at ~100 ng/mL and an almost 10-fold difference between the animals (**Fig 4D**). These results suggest n-PGT121 shows limited improvement on PGT121 delivery in Group II animals with chronic infection, in contrast to the prior results on acute infection.

### Nanocapsules significantly impact SHIV infection in infant macaques with acute infection

**Fig 5:**
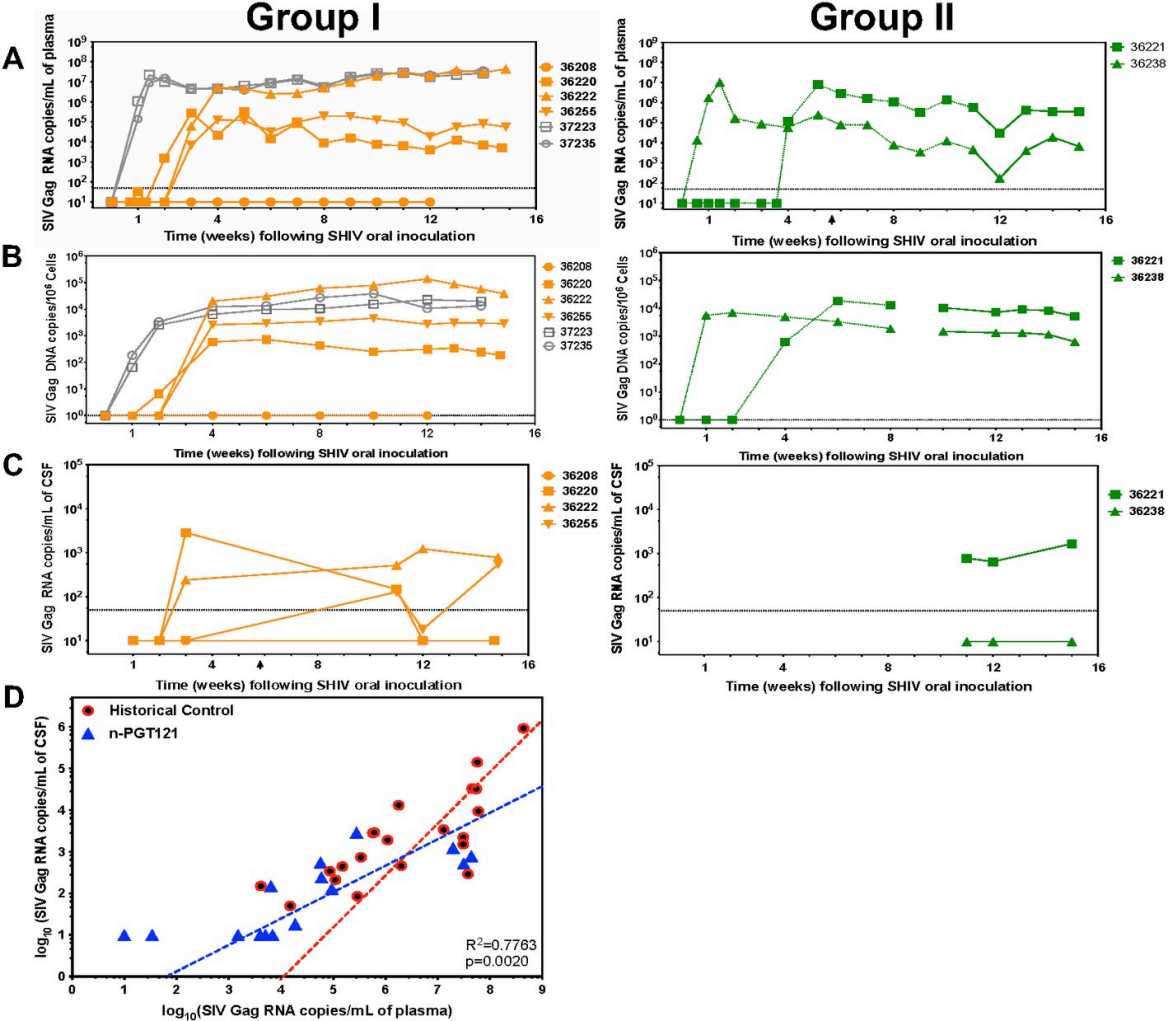
Nanocapsules reduce viremia in both plasma and CNS of SHIV-infected infant rhesus macaques. A) Plasma viral loads (viral RNA copies), B) the PBMC-associated viral loads (vDNA), and C) the CSF viral loads (viral RNA copies) were measured longitudinally in n-PGT121 treated animals in the acute infection group (Group I, n = 4) and chronic infection group (Group II, n = 2). Key to individual animals is shown. D) The comparison between Historical Control and n-PGT121 animals on correlation between viral RNA copies in plasma and CSF. Each dot represents one animal, and all data are fitted by nonlinear log-log line regression. The significance between the correlations of two groups is calculated with a multiple regression model. A statistically significant difference between slopes is observed in the multiple regression model (p-value = 0.0020). Both untreated and treated animals with native bNAbs or bNAbs+cART are combined and plotted as Historical Control (Untreated and Treated in Fig 2B).

To study the virologic outcomes of n-PGT121 treatment, we followed the animals for 12–15 weeks after SHIV infection and quantified VL in plasma and vDNA in PBMCs. Viral RNA copies were quantitively compared between untreated and Group I animals (**Fig 5A**). Contemporaneous untreated control animals were infected with the same stock and dose of virus (Animals 36206 and 36207) as part of another study in infants, and these showed 2–5×10^7^ peak VL in plasma and vDNA in PBMCs and were euthanized at week 2{Shapiro, 2020 #48}. Two additional untreated controls (Animals 37223 and 37235) were infected with the same stock and dose of virus for this study and showed a peak VL at 10^7^ copies/mL and maintained this level through the study, consistent with other control infants infected with this stock of virus at this dose and age. As reported in our previous publication, 50% of infected animals, which were treated with a cocktail of PGT121 and VRC07-523 (5 mg/kg) with four doses initiated at Day 2, showed breakthrough viremia around 3 weeks after the last injection [48]. One of the four animals (Animal 36208) maintained undetectable levels of plasma VL for the duration of the study, while viremia in the other 3 animals was delayed for 2 weeks—comparable to the rebound kinetics of published bNAb cocktail-treated animals. Moreover, 2 out of 3 rebound animals suppressed their VL by a factor of 3 log_10_ compared to untreated animals. The impact of n-PGT121 on cell-associated viremia in PBMCs is shown in **Fig 5B**. SHIV-1 cell-associated vDNA in PBMCs correlates with plasma viremia. Similar to results on plasma VL in Group I, the animal (Animal 36208) fully suppressing plasma VL had no detectable vDNA; meanwhile, the three viremic animals reached stable cell-associated vDNA levels at Week 4 post-infection. We next evaluated the impacts of n-PGT121 on CNS infection in these Group I animals (**Fig 5C**). The CSF VL of all four animals was negative at Week 2, with two of them remaining undetectable at the next collection time point (Week 3). However, three of the animals became positive by Week 11, at which point all three viremic animals were treated with a second infusion of n-PGT121. Two of them showed a response and fully suppressed CSF VL after the second treatment. At necropsy at Week 15, two animals (Animals 36255 and 36222) with high plasma VL of >10^5^/mL and >10^7^/mL, respectively, showed detectable CSF VL. The suppression efficacy by n-PGT121 on CSF VL negatively correlates with plasma viremia setpoints: the CNS infection can be fully suppressed by n-PGT121 when plasma VL is <10^4^ copies /mL. The animal (36220) with undetectable VL in plasma was negative for vDNA in brain tissue. Of the three viremic animals, one animal (36255) showed detectable but <1 vDNA copy in 10^6^ brain cells (**S3 Table**). To concentrate the infected cells and accurately detect the infection in the brain, we isolated microglia and detected microglia-associated vDNA in one more animal (Animal 36222).

The same studies were also applied to Group II animals with chronic SHIV infection, animals that were infected as part of the SHIV stock titration study. In contrast to Group I, Group II animals with chronic infection showed an approximately 1.5-log decline in plasma VL within the first week after n-PGT121 treatment, then were assessed for rebound VL back to pre-treatment levels in the following weeks (**Fig 5A**). Treatment in Group II did not show an impact on vDNA in PBMCs (**Fig 5B**). Moreover, the CSF VL of Group II animals showed the same trend as Group I: one animal with plasma VL of ~5×10^3^ copies /mL had undetectable CSF VL through the entire study, while the other with a plasma VL of >10^5^/mL did not show any suppression in CSF VL after the treatment at Week 11 (**Fig 5C**). Cell-associated vDNA was also detected in brain tissues from Group II animals. One animal (Animal 36238) showed positive vDNA copies in brain cells, while the other one (Animal 36221) had detectable microglia-associated vDNA in microglia isolated from brain tissues (**S3 Table**).

To further evaluate the impact of n-PGT121, we analyzed the correlation between plasma VL and CSF VL in n-PGT121 animals at all time points when both were detectable (**Fig 5D**). Because correlations of the plasma VL and CSF VL between untreated animals and animals with native bNAb-based therapy were comparable, we were able to combine all VL data and use them as the historical controls. Compared to the historical controls, the plasma-CSF VL correlation of n-PGT121-treated animals showed a striking and statistically significant shift towards lower CSF VL at a given plasma VL (*p* = 0.0020). We also plotted the relationship between vDNA in microglia and either the plasma VL or CSF VL in Group I and Group II; however, it could not be statistically analyzed due to the low sample numbers in each group (**S5 Fig)**. Taken together, these results indicate the enhanced levels of PGT121 in the CNS delivered by nanocapsules result in a more pronounced effect on CNS infection.

### Impact of bNAb nanocapsules on tissue-associated viremia is consistent with that of plasma viremia

**Fig 6:**
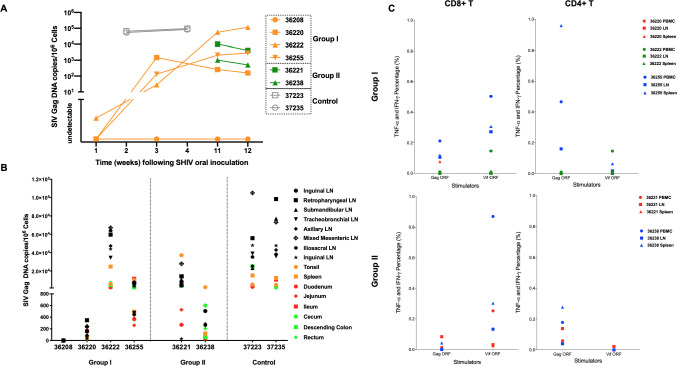
Impact of bNAb nanocapsules on tissue-associated viremia is consistent with plasma viremia. A) Cell-associated viral loads (vDNA) were measured in lymph node biopsies longitudinally in untreated control, Group I, and Group II animals. B) SHIV DNA was quantified by ultrasensitive nested qPCR in all tissue samples from the untreated control, Group I, and Group II animals at necropsy. Tissues are color coded in the figure. Black symbols represent lymph nodes from different locations. Red symbols represent tissues in the small intestine. Green symbols represent tissues in the large intestine. Orange symbols represent the other lymphatic tissues, including tonsil and spleen. C) CD8+ and CD4+ T cell responses to SIVmac239 Gag ORF 15-mer peptide pool, and SIVmac239 Vif ORF 15-mer peptide pool were measured by flow cytometric intracellular cytokine staining (ICS). Cells without stimulator were used as negative controls. Different tissue types (PBMC, LN from mesentery, spleen) are indicated with individual symbols as shown and the animal number is indicated next to the symbol.

To evaluate the effect of n-PGT121 on viral seeding in tissues, we monitored vDNA copies in inguinal LN biopsies (**Fig 6A**) and lymphoid and gastrointestinal (GI) tract tissues at necropsy (**Fig 6B**). As shown in **Fig 6A**, 10^5^ of vDNA in inguinal LNs was observed at Week 2 post-infection and remained stable by Week 4 in untreated animals. In Group I, the animal (Animal 36208) with no plasma viremia showed undetectable vDNA in all LN biopsies throughout the study. Meanwhile, three viremic animals showed detectable vDNA in inguinal LNs at Week 3 with 100- to 10000-fold lower levels than those of controls at Week 2; additionally, two animals (Animals 36222 and 36255) showed increasing vDNA at Week 11 right before the second treatment. However, vDNA copies of all three viremic animals did not decrease after the second treatment. On the other hand, Group II animals showed a 1-log_10_ decrease of vDNA in inguinal LNs with n-PGT121 treatment at Week 11. These results show n-PGT121 treatment at early infection prevents viral seeding and reservoir formation; however, the impact of n-PGT121 became limited once viral reservoirs were established in tissues.

In order to map vDNA distribution in main reservoir-harboring tissues, we sampled a comprehensive panel of up to 16 tissues at necropsy, including lymphoid tissues (tonsil, lymph nodes from eight locations, and spleen) as well as GI tract tissues (duodenum, jejunum, ileum, cecum, descending colon, and rectum) (**Fig 6B)**. Consistent with the plasma VL and vDNA in LN biopsies, the animal (Group I, Animal 36208) with undetectable plasma VL showed no detectable tissue-associated virus in all tissues, consistent with prevention of infection or clearance of SHIV. Both Group I and Group II animals significantly decreased VL harbored in all collected tissues compared to control animals (Group I: *p*<0.0001; Group II: *p* = 0.0008). Furthermore, the suppression by n-PGT121 was superior in lymphoid tissues compared to in GI tract tissues (lymphoid: *p* = 0.0003; GI tract: *p* = 0.0010), in accordance with our previous publication of heightened delivery by nanocapsules into lymphoid tissues over GI tract tissues [64].

To evaluate T cell immunity in the SHIV-infected animals treated with n-PGT121 during acute and chronic infection, we used flow cytometric intracellular cytokine staining (ICS) to measure specific responses (responder: TNF-α+ and IFN-γ+) by CD8+ and CD4+ cells in LN, PBMC, and spleen to the SIV proteins Gag and Vif (**Fig 6C**). All response values were background subtracted, where cells without stimulators were used as backgrounds. The animal (Animal 36208) without plasma VL showed undetectable T cell responses and was excluded from this figure. Viremic animals in both groups developed both CD8+ and CD4+ responses to HIV Gag and Vif. The CD8+ T cells, especially in PBMC, showed higher TNF-α+ and IFN-γ+ percentages to HIV Vif than HIV Gag. In contrast, the CD4+ T cells, especially in spleen, had superior responses to HIV Gag.

## Discussion

Overall, our results demonstrate a novel nanotechnology approach utilizing nanocapsules to deliver bNAbs into the CNS, which is an important anatomical compartment harboring productive and latent virus. Systemically injected nanocapsules prolong the effective concentration of bNAbs in blood and enhance their penetration and persistence into the CNS in a model for SHIV in newborn and infant macaques. The persistently high levels of viremia that this strain of SHIV induces in untreated newborns provides a stringent model to test the CNS delivery concepts. In the Group I study demonstrating treatment during acute infection, a single dose of encapsulated PGT121 (n-PGT121) at 48 hours post-infection resulted in improved SHIV control, reduced tissue viral seeding, and decreased pathogenesis. Moreover, one of four infected infant NHPs was cleared from infection (**Fig 5A** and **5C**) and failed to seroconvert. Empirically, n-PGT121 shows a more pronounced effect on CNS infection by enhancing the delivery of PGT121 in the CNS.

The establishment of cell-associated vDNA in PBMCs within 2–3 weeks and lack of vDNA decrease with the later treatment after reservoir seeding suggests the ineffectiveness of bNAbs against established reservoirs (**Fig 5B**). Furthermore, we observed higher HIV-specific CD8+ T cell response to HIV Vif, while CD4+ T cells only responded to HIV Gag (**Fig 6C**). Virus rapidly disseminates and seeds in tissue reservoirs after infection; therefore, the therapeutic effect of bNAbs during the acute infection stage depends on the speed and extent of their biodistribution to the viral replication sites [47]. Thus, by improving both maintenance and tissue penetration, one systemic injection of nanocapsules shows suppression of SHIV comparable to that of four injections of native bNAb cocktail, and more importantly, greater control of SHIV replication in the CNS.

The efficient delivery of bNAbs to the CNS is attributed to the special properties of the nanocapsules. The bNAb nanocapsules have a uniform particle size of 20–30 nm and are thought to penetrate the BBB via transcytosis rather than through tight junctions. Choline transporters (ChTs) and nicotinic acetylcholine receptors (nAChRs) are widely expressed in the nervous system and luminal brain capillary endothelial cells. As such, the monomer used to synthesize nanocapsules, 2-methacryloyloxyethyl phosphorylcholine (MPC), contains analogues of acetylcholine (ACh) and choline (Ch) specifically to interact with ChTs and nAChRs [66]. BBB penetration of nanocapsules can be inhibited with the presence of soluble ACh, Ch, α-bungarotoxin (α-BTX), an irreversible inhibitor for nAChRs, and hemicholinium-3 (HC3), an inhibitor for ChTs, indicating that ChTs and nAChRs facilitate the nanocapsules transcytosis [65,66]. Moreover, prolonging the half-life of the nanocapsules increases the interaction possibility between nanocapsules and receptors on endothelial cells of BBB, including nAChRs and ChTs.

We demonstrate here that nanocapsules have a pronounced effect on CNS infection. We expect to further improve suppression of the CNS infection through two strategies. First, bNAb effectiveness in the CNS can be improved by increasing bNAb levels in the CNS. The bNAbs eliminate HIV-1 virions by direct neutralization in any location harboring free virions, including the CNS. Moreover, the mechanism by which bNAbs decrease viral rebound is likely to be the clearance of infected cells via complement-dependent cytotoxicity (CDC) and/or antibody-dependent cellular cytotoxicity (ADCC) in patients. The human bNAb PGT121 we tested is an IgG1, and its Fc has been shown to bind well to rhesus Fc-γ RIII [79]. Although both CDC and ADCC processes function in the CNS [80–85], their functionality is limited due to insufficient bNAb levels in CNS. Therefore, by increasing the antibody levels in the CNS, nanocapsules demonstrate their capability to decrease CNS VL. We can further tune nanocapsules to achieve higher bNAb delivery into the CNS over current formulations through the appropriate selection and optimization of three key components: 1) zwitterionic monomers that form polymer structures and affect biodistribution; 2) crosslinker selections for the responsive release of cargo; and 3) ligand conjugation on the surface of nanocapsules for targeting to desired tissues. By optimizing these aspects of the nanocapsules, the greatest amount of bNAbs can be delivered to the CNS to maximize viral suppression. Second, bNAb combinations delivered by nanocapsules can prevent virus escape. One critical issue with using bNAbs for therapeutic purposes is the emergence of viral escape mutants against single mAbs [86,87]. Fortunately, nanocapsules can be universally applied to various bNAbs targeting multiple different epitopes on the HIV envelope. By synthesizing nanocapsules with the same formulation as n-PGT121, all nanocapsules, regardless of the bNAb cargo, are expected to show the same pharmacokinetics, biodistribution, and brain delivery efficiency. Therefore, we can easily prepare combination treatments of bNAb nanocapsules, including VRC07-523, 10–1074, PG16 and 3BNC117, previously tested in humanized mouse and monkey models to prevent viral escape mutants [36–38].

Besides improved suppression on CNS infection, we also observed 100 to 1,000-fold decrease in LN vDNA by n-PGT121 treatment at the acute phase of infection. We have previously confirmed the improved delivery into LNs by nanocapsules encapsulating RTX with the same formulation in NHPs [64]. Our results show that a single intravenous injection of n-RTX results in 2-3-fold greater levels in the LNs of RTX at Week 3 post-injection. Although it is difficult to confirm the improved levels in LNs with n-PGT121 due to the limited historical data on native PGT121, we ascribe improved suppression of viral DNA to this enhanced antibody delivery in LNs.

Although the effect of n-PGT121 was insufficient in Group II animals with chronic infection, the treatment achieved transient <2-log decrease in the plasma VL and no impact on the CSF VL (**Fig 5A** and **5C**), we gained some insights by comparing between the two groups. Compared to Group I, Group II animals showed ~ three-fold lower initial PGT121 concentrations in both plasma (**Fig 4B**) and the CSF (**Fig 4D**). The low bNAb concentrations could be attributed to more free virions and infected cells in chronically infected animals. An average CSF concentration maintained 0.75% of the plasma concentration, which was in accordance with our observation that the brain delivery effectiveness of this nanocapsule system is plasma concentration-dependent [66]. Even though bNAbs demonstrate their potency to suppress virus replication, their effect on the established reservoirs is not fully understood [45]. Our finding showing failure to reduce cell-associated vDNA with n-PGT121 supports the concept that the PGT121 is ineffective in eliminating established reservoirs (**Fig 5B**). Moreover, it has been demonstrated in patients that PD-1 expression was upregulated on CD8 and CD4 T cells in people with chronic HIV infection naïve for anti-retroviral therapy, which indicates impaired T-cell function and inability of HIV-specific cellular immune responses to effectively control viremia [88]. In this study, Group II animals infected for 11 weeks and naïve for any treatments before n-PGT121, comparable to chronically infected patients, showed significantly lower HIV-specific T cell responses in T cells isolated from blood, LNs, and spleens than that in Group I with acute infection (**Fig 6C**). Therefore, T cell exhaustion under chronic infection may also contribute to the insufficient and transient suppression by n-PGT121 in Group II animals.

Though HIV-1-specific bNAb sequences were derived from humans, bNAbs still have the potential to stimulate an immune response in recipients and induce anti-drug antibodies (ADA). The development of ADA results in impaired action and decreased pharmacokinetic profiles of the bNAbs [89]. Moreover, the development of ADA to therapeutic protein drug products may induce harmful responses in patients [90]. In our published studies, we observed that clearance of bNAbs was in conjunction with the development of robust ADA responses in 3 weeks after bNAb injection [47]. Studies using adenovirus-associated virus (AAV) to deliver genes expressing bNAbs in SHIV infected macaques showed host ADA responses against bNAbs within 4 weeks after injection and maintained the response levels for over 20 weeks, which resulted in a rapid elimination of bNAbs within 3–4 weeks after bNAb titers peaked [91,92]. In our published studies, we demonstrated that the polymer shell of a nanocapsule shields the encapsulated foreign protein from the host immune system and significantly prevents the production of IgG, IgE and antigen-specific IgG [63]. Therefore, we observe a longer half-life of n-PGT121 than native PGT121 (**Fig 4B**). Although PGT121 is protected in nanocapsules and controlled released from nanocapsules maintains an effective and stable concentration, ADA may still develop against the foreign IgG of PGT121 once PGT121 is released. Thus, ADA response is likely responsible for a lower concentration and shorter maintenance seen following the second treatment of n-PGT121 in Group I animals, implying a limited impact on virus suppression with the repeated treatment.

To conclude, current bNAb therapies are insufficient to treat CNS infection as they cannot achieve adequate CNS delivery blocked by the BBB. Our nanocapsule technology provides a non-invasive means to suppress CNS infection by prolonging the half-life and improving the delivery of therapeutic bNAb into the CNS.

## Materials and methods

### Ethics statement

Mouse studies were performed at University of California, Los Angeles (UCLA) in Los Angeles, CA, USA in compliance with all ethical regulations for animal testing and research. The animal housing, handling, and study protocol follow the guidance of the Guide for the Care and Use of Laboratory Animals and the U.S. Public Health Service Policy on the Humane Care and Use of Laboratory Animals and was approved by the UCLA institutional animal care and use committee (IACUC). Non-human primate studies were performed at the Oregon National Primate Research Center (ONPRC) in Beaverton, OR, USA in compliance with all ethical regulations for animal testing and research. The ONPRC is accredited by the American Association for the Accreditation of Laboratory Animal Care (AAALAC) International and adheres to the Guide for the Care and Use of Laboratory Animals and the U.S. Public Health Service Policy on the Humane Care and Use of Laboratory Animals. The study protocol was approved by the Oregon Health & Science University (OHSU) West Campus Institutional Animal Care and Use Committee (IACUC).

### Animal model

For viral exposure and treatment studies in one-month-old infant rhesus macaques (*Macaca mulatta*), 6 macaques were obtained from the breeding colony at one week of age and raised in the ABSL-2 infant nursery for 3 weeks, during which they were adapted to formula feeding. Infants were then transferred to ABSL-2+containment for study procedures involving SHIV challenge. Animals were randomly assigned to study groups as they were born and were excluded from the study if the animal, or its sire or dam, could not be confirmed negative for Mamu-B*08 and -B*17 MHC Class I alleles, which are associated with spontaneous lentiviral control. Animals were housed in age-matched pairs throughout the study and were monitored for clinical signs of disease by regular evaluation of body weight, peripheral lymph node size, appetite, behavior, and stool quality. Animals were euthanized under IACUC guidelines using standard methods consistent with the recommendations of the American Veterinary Medical Association (AVMA) Guidelines for Euthanasia.

### SHIV virus challenge

The SHIV_SF162P3_ stock was prepared based upon our previous protocol [47]. Spleens and blood from naïve rhesus macaques were processed to generate single cell suspensions, which were cryopreserved in the liquid nitrogen gas phase. To generate a stock of SHIV_SF162P3_ for use in animals, a small-scale virus expansion was first performed using SHIV_SF162P3_ stock obtained from the NIH AIDS Reagent Program (catalog number 6526) as inoculum; the day 7 supernatant of this small culture was subsequently used to inoculate a large-scale culture for virus stock production. CD4 cells were enriched from rhesus PBMCs by NHP CD4 MicroBeads (Miltenyi) then pooled and activated for 24 h in R15-100 media (RPMI1640, 15% FBS, 100 U/ml IL-2) containing a stimulation cocktail of Staphylococcal enterotoxin B (2 μg/ml, Toxin Technologies) and antibodies against CD3 (300 ng/ml, clone CD3-1, Mabtech), CD28 (1.5 μg/ml, clone L293, BD Biosciences), and CD49d (1.5 μg/ml, clone 9C10, BD Biosciences). A total of 2×10^7^ cells were spinoculated for 2h at 1600×g at room temperature with 50μL of SHIV_SF162P3_ virus. Cells were washed the next day to remove free virus, and cultures were maintained by refreshing half of the media every other day until supernatant was harvested and banked on day 7. TCID_50_/ml was measured in rhesus PBMCs using an assay adapted from Ranajit Pal (ABL, personal communication). Briefly, the viral stock was serially diluted 4-fold in a 96-well plate, beginning with a 1:2 dilution, making seven replicates per dilution step. Viral dilutions were incubated with 2×10^5^ PHA-stimulated naïve rhesus PBMC per well for 7 days at 37°C under 5% CO_2_. Supernatants were then harvested and the presence or absence of virus in each well was determined by SIV p27 ELISA (RETROtek SIV p27 Antigen ELISA, ZeptoMetrix Corporation, Buffalo, NY). The positive cutoff value was determined by dividing the average OD_450_ of the lowest SIV p27 standard in the ELISA plate (63 pg/ml) by 2 and adding 0.05. TCID_50_ was calculated using the Spearman-Karber method. The stock was titered in vivo in infant rhesus macaques, which showed consistent high viremia at 2 mL {Shapiro, 2020 #48}. Animals received a total of 2 ml (4.1×10^4^ TCID_50_ measured in rhesus PBMC) of undiluted cell-free virus by swallowing two 1 ml doses 15 min apart. Virus aliquots were transported on dry ice and thawed at room temperature or in hand just prior to animal challenge.

### Synthesis of broadly neutralizing antibody (bNAb) nanocapsules

The IgG1 antibody PGT121 was produced by transient transfection in Expi293F cells (Thermo-Fisher Scientific, Inc.) and affinity purified on Protein A columns to >95% purity. All chemicals were purchased from Sigma-Aldrich (St. Louis, MO, US) unless otherwise noted. All cell culture reagents were purchased from ThermoFisher Scientific (Waltham, MA) unless otherwise noted. Hydrolysable crosslinker Poly(DL-lactide)-b-Poly(ethylene glycol)-b-Poly(DL-lactide)-diacrylate triblock (A102, PLA-PEG-PLA) was purchased from PolySciTech Akina, Inc (West Lafeyette, IN). PGT121 were encapsulated via *in-situ* polymerization technology. The PGT121 (1 mg/mL in PBS) was mixed with 2-methacryloyloxyethyl phosphorylcholine (MPC) as monomer (40% m/v in PBS) and PLA-PEG-PLA (10% m/v in PBS) as well as glycerol dimethacrylate (GDMA, 10% m/v in DMSO) as degradable crosslinkers. Then the polymerization was initiated by adding ammonium persulfate (APS, 10% m/v in PBS) and N, N, N’, N’–tetramethylethylenediamine (TEMED) solution. Synthesized nanocapsules were dialyzed against PBS and purified by passing through a hydrophobic interaction column (Phenyl-Sepharose 4BCL).

### PGT121 nanocapsule (n-PGT121) kinetics and biodistribution in mice

Kinetics and biodistribution of native PGT121 and n-PGT121 were determined by monitoring the free PGT121 concentration in animal body fluids and perfused tissue homogenates. Briefly, C57BL/6 mice were randomly divided into two groups and injected retro-orbitally at a dose of 5 mg/kg/mouse. CSF was collected from a mouse under anesthesia by ketamine and xylazine (100 mg/kg each). After CSF collection, this mouse was perfused with cold PBS and euthanized, then organs were harvested. All perfused tissues were homogenized by vortexing with ceramic beads in PBS containing protease inhibitor cocktail. The method used to measure the PGT121 concentration is described in “antibody concentration determination in plasma and CSF” under the section “PGT121 nanocapsule (n-PGT121) therapy in infected infant rhesus macaques”.

### Evaluation of BBB integrity by Evans blue (EB) dye extravasation in mice

C57BL/6 mice were randomly divided into three groups and injected withnative PGT121 and n-PGT121 retro-orbitally at a dose of 5 mg/kg/mouse. One day after treatment, EB dye was injected through tail vein and allowed to circulate for 1 h, and perfusion was performed to remove dye from the circulation. Afterwards, brains were collected, dissected, and homogenized for fluorescent intensity measurement at 620/680 nm. The absorbance values were converted into ng dye/mg tissues using a standard curve of EB in ethanol.

### PGT121 nanocapsule (n-PGT121) therapy in infected infant rhesus macaques

Animals in Group I and Group II were given one dose of n-PGT121 (5mg/kg) intravenously at 48 h or Week 11 after SHIV exposure.

#### Blood and tissue harvest and processing

Peripheral blood of was collected into EDTA blood collection tubes prior to virus exposure on the day of challenge, and weekly thereafter. Blood tubes were centrifuged at 1850 rpm for 25 min at 4°C with the brakes off in order to separate plasma from cells. The plasma supernatant was pipetted off and aliquots were stored at -80°C. The remaining blood fraction was resuspended in sterile PBS to double the original volume, and PBMC were isolated by centrifugation in SepMate tubes (StemCell Technologies) over Lymphocyte Separation Medium (Corning). For vDNA detection by quantitative PCR as described below, 3 × 10^6^ PBMCs were pelleted at ~20,000 × g in a benchtop microcentrifuge and frozen at −80°C; any remaining PBMCs were cryopreserved in liquid nitrogen. Inguinal or axillary lymph nodes were biopsied at various times post-exposure, and single cell suspensions were made by crushing the tissue through a 100 μm strainer. Lymph node cells were then frozen as pellets of 3 × 10^6^ cells at −80°C for viral quantitation, and any remaining cells were cryopreserved in liquid nitrogen. At necropsy, blood, cerebrospinal fluid (CSF), and a panel of up to 16 solid tissues were harvested. CSF was collected into a 2 ml vial and stored at −80°C. From each solid tissue, 100 μg samples were excised and frozen at −80°C in 2 ml tubes pre-filled with 1.4 mm zirconia beads (Spex SamplePrep) to facilitate tissue homogenization with a bead beater, nucleic acid extraction, and vDNA detection by quantitative PCR.

#### Antibody concentration determination in plasma and CSF

PGT121 levels in plasma and CSF were quantified using plates coated with ST0A9 [93] to measure PGT121 or with HIV_SF162_ envelope gp140 to measure Envelope antibodies (seroconversion). Nunc MaxiSorp (ThermoFisher) plates were coated overnight with 200 ng/well of ST0A9 in PBS, washed with PBST (0.1%Tween 20/PBS) five times, and blocked with PBST with 2% BSA for 1 h at room temperature. Serial dilutions of all samples were plated in duplicate. Plasma was incubated for 1 h at room temperature, followed by a PBST wash. Bound bNAbs were probed with a horseradish peroxidase-labeled goat anti-human IgG (1:5000 dilution; Jackson Laboratories) for 30 min at room temperature. The plate was washed and 3,3’,5,5’-tetramethylbenzidine (TMB, Pierce) substrate was added. Once color was developed stopping buffer was added and the optical density at 450 nm was read. GraphPad Prism and Microsoft Office software were used to plot standard curves and calculate monoclonal or polyclonal antibody concentrations.

#### Microglia isolation

Microglia were isolated from brain tissues from different locations as described in the published study [94]. Brain tissues were perfused and minced, and then dissociated in the Dispase DNase Papain (DDP) solution (Dispase II: 1.2 U/mL; Papain: 1 mg/mL; DNase I: 20 U/mL) on rotating racks for 30min. Dissociated brain tissues were mashed against 40 μm cell strainer to collect single cells. Single cells were resuspended in 70% percoll and separated by the percoll gradient (top to bottom: 1×HBSS, 30% percoll, 37% percoll, and 70% percoll). Cells were centrifuged under the percoll gradient setup for 40 min at 300×g without brake. The cell layer between 37% and 70% percoll contained microglia. Isolated microglia were washed and stained with CD11b and CD45 to confirm the purity by flow cytometry. Anti-CD11b (Clone M1/70) and anti-CD45 (Clone MEM-55) were purchased from BioLegend, Inc (San Diego, CA, US).

#### Viral nucleic acid detection in plasma, cells, and tissue homogenates

Nucleic acids from plasma, CSF, microglia, peripheral blood mononuclear cells (PBMC), and lymph node biopsy pellets were purified using a Maxwell 16 instrument (Promega, Madison, WI) per the manufacturer’s protocol with the LEV Viral Nucleic Acid Kit for plasma and CSF and the LEV Whole Blood Nucleic Acid Kit for cells. SHIV viral RNA in plasma and CSF was measured by quantitative RT-PCR with a detection limit of 50 copies/ml using a method developed by Cline et al. [95] with minor modifications to the master mix to increase sample input. SHIV vDNA in cellular DNA from microglia, PBMC or lymph node biopsy pellets was measured using quantitative PCR with Fast Advanced Mastermix on an Applied Biosystems QuantStudio 6 Flex instrument (Life Technologies, Carlsbad, CA). Reactions were performed with 2 μg nucleic acid input for 45 cycles using the FAST-cycling protocol (95°C for 1 s, 60°C for 20 s) in a 30 μl reaction volume. Virus copy numbers were estimated by comparison to a linearized pBSII-SIVgag standard curve and calculated per cell equivalent using the input nucleic acid mass and by assuming a DNA content of 6.5 μg per 10^6^ cells, with a detection limit of 2 copies/μg DNA or 10 copies/10^6^ cells. Primers and probe used for plasma and PBMC assays were those described by Cline et al. [95]: SGAG21 forward (GTCTGCGTCATPTGGTGCATTC), SGAG22 reverse (CACTAGKTGT CTCTGCACTATPTGTTTTG), and pSGAG23 (5’-(FAM)-CTTCPTCAGTKTGTTTCACTTTCTCTTCTGCG -(BHQ1)-3’). vDNA was measured in necropsy tissue samples using an ultrasensitive nested quantitative PCR assay44 targeting a highly conserved region of gag in SIV and SHIV with a detection limit of 0.02 copies/μg DNA or 1 copy/10^7^ cells. Primers used for pre-amplification of vDNA were SIVnestF01 (GATTTGGATTAG CAGAAAGCCTGTTG) and SIVnestR01 (GTTGGTCTACTTGTTTTTGGCATAGTTTC), and primers for quantitative PCR were SGAG21 forward, SGAG22 reverse, and pSGAG23 as described above. Samples were heated at 95°C for 5 min and then put on ice. Each sample was assayed in 12 replicates of 5 μg each. In order to assess PCR reaction efficiency, 10 copies of DNA containing the SIV gag target sequence were spiked into two of the reactions. None of the tested DNA samples showed significant amplification inhibition, defined as a 5-cycle delay relative to the amplification kinetics of reactions containing solely 10 copies of standard. The first round of amplification was performed in 12 cycles (95°C for 30 s, 60°C for 1 min) in a 50 μl reaction volume. Reactions were performed for 45 cycles using the FAST-cycling protocol described above in a 30 μl reaction volume. Virus copy numbers were calculated from the frequency of positive replicates using the Poisson distribution and expressed as copies per μg DNA, or as copies per cell equivalent by assuming a DNA content of 6.5 μg DNA per 10^6^ cells. Staff members performing the viral RNA and DNA assays were blinded to the experimental groups and conditions for all samples tested.

#### Viral outgrowth assay to measure inducible virus in isolated microglia

To measure inducible replication-competent virus in tissues, a TZA assay was used as described [47] with the following modifications. Microglia were stimulated *in vitro* for 5 days in R15-100 media (RPMI1640, 15% FBS, 100 U/ml IL-2) with M-CSF (20ng/mL) and IL-10 (25ng/mL). On Day 3 of stimulation, half of the cell culture supernatant volume was removed and replaced with fresh media. On the morning of the assay, 1 × 10^4^ TZM-bl cells were plated into each well of a 96-well culture-treated flat bottom assay plate and allowed to adhere for 4–6 h. Stimulated microglia samples were then plated in quadruplicate on top of the TZM-bl cells in a fourfold dilution series starting with 2.5 × 10^5^ cells/well. To detect the presence of infectious virus in each well, Bright-Glo (Promega) was added after 48 h and luciferase activity was read out on a luminometer. Wells were considered positive for viral outgrowth if the luciferase signal exceeded the average plus 3 standard deviations of 12 replicate wells containing only TZM-bl cells (NIH AIDS Reagent Program, catalog number 8129). The number of infectious units per million CD4+ cells (IUPM) in each sample was calculated using the IUPMStats v1.0 Infection Frequency Calculator available at http://silicianolab.johnshopkins.edu/.

#### T cell responses by flow cytometric intracellular cytokine staining (ICS)

SIV-specific CD4+ and CD8+ T cell responses in blood, lymph nodes, and spleens were tested by flow cytometric ICS assay, as described in detail [96]. Briefly, isolated CD4+ and CD8+ T cells were incubated with antigen peptides (SIVmac239 Gag ORF 15-mer peptide pool and SIVmac239 Vif ORF 15-mer peptide pool) and the co-stimulatory molecules CD28 and CD49d (BD Biosciences) for 1 hour, followed by addition of brefeldin A (Sigma-Aldrich) for an additional 8 hours. Cells incubated with Concanavalin A (ConA, Sigma-Aldrich) were used as positive controls, while cells without antigen stimulation were used as negative controls. Stimulated cells were fixed, permeabilized, and stained. The flow cytometric analysis was performed on an LSR-II instrument (BD Biosciences). Analysis was done using FlowJo software (Tree Star). In all analyses, gating on the light scatter signature of small lymphocytes was followed by progressive gating on the CD3+ population and then the CD4+/CD8- versus CD4-/CD8+ T cell subsets. Antigen-specific response frequencies for CD4+ or CD8+ T cell populations were determined from intracellular expression of TNF-α and IFN-γ.

### Statistical analysis

For analysis of correlations between plasma VL and CSF VL, CSF VL and vDNA in brain tissue, and plasma VL and vDNA in brain tissue (Figs 1A, 1B, and S1), nonlinear log-log line regression or nonparametric correlations were used, due to the skewed distribution of the data. The significance between two correlations of plasma-CSF VL was calculated by multiple regression model between slopes (Figs 2B and 5D). The virus distribution in different locations of the brain (occipital, parietal, temporal, and cerebellum) was compared to frontal by one-tailed unpaired t-test with Welch’s correction (Fig 1C). The comparison between plasma VL in untreated and bNAb-treated animals (Fig 2A) as well as PGT121 and n-PGT121 concentrations in different tissue compartments (Figs 3C, 3D, and S4) were calculated by two-tailed unpaired t test. Half-life estimation (Figs 3B and 4B) was calculated by the one phase exponential decay model. Statistical analyses were performed in GraphPad Prism 7 or in SAS 9.4.

## Supporting information

S1 Fig Correlation between SHIV_SF162P3_ Gag RNA copies in plasma and vDNA copies in brain tissue. The animals showing both positive RNA copies in plasma and vDNA are included in the figure. Each dot presents one animal, and all data are fitted by nonlinear log-log line regression. R^2^ and p value indicate the fitness of Pearson correlation. Dotted lines indicate limits of detection.
(DOCX)

S2 Fig Purification of isolated microglia from the brain tissue. The brain tissues of rhesus macaques were dissociated by papain, Dispase II, and DnaseI treatment, and followed by Percoll gradient sedimentation. Microglia (62%) were identified as CD45^low^ CD11b+ cells, and macrophages (4%) were gated as CD45^high^ CD11b high cells.
(DOCX)

S3 Fig Structure, morphology, and sustainable release of PGT121 nanocapsules (n-PGT121). A) The release kinetics of nanocapsules in PBS.10 μg of n-PGT121_intact_, n-PGT121, and n-PGT121_hydrolysable_ were added into 1 ml PBS, respectively, and incubated at 37°C for 3 week. The concentration of released PGT121 was measured by ELISA. The concentration standard curve for ELISA was prepared with native PGT121. B) Nanocapsulation of PGT121 improves levels of its tissue penetration, including CNS, in rats. A single dose (10 mg/kg) of native and encapsulated PGT121 was administered in rats through tail-vein injection (n = 2). The concentrations of free PGT121 in plasma, CSF, and brain on Day 7. Brain tissues were collected from perfused animals, homogenized in PBS (1 mg tissue in 100 μl PBS), and tested by ELISA. C) Size of native PGT121 and n-PGT121 detected by dynamic light scattering (DLS). D) Representative transmission electron microscopy (TEM) image of n-PGT121. E) Evaluation of BBB leakage using evans blue (EB) in the brain tissues from mice treated with PBS, PGT121, and n-PGT121 one day post-injection. Brain tissues were harvested from mice after perfusion and homogenized for EB dye detection. Mice bearing brain tumors were used as positive control. EB dye fluorescence intensity was detected at 620/680 nm. Dye leakage was calculated from absorbance values to ng dye using a standard curve of EB in ethanol.
(DOCX)

S4 Fig The nanocapsules improve PGT121 concentration in rhesus macaque CSF compared to native PGT121. A) 10mg/kg of bNAb cocktail (5mg/kg PGT121 and 5mg/kg VRC07) was administered in rhesus macaques as the control group. Plasma and CSF were **collected** on Day1 and Day7 after infusion. The concentration of PGT121 in plasma and CSF was measured by ELISA in duplicates. Each symbol represents one individual rhesus macaque. B) The percentages of PGT121 CSF-concentration of plasma-concentration from infant rhesus macaques treated with native PGT121 on Day1 and Day7 after injection. There was no statistically significant difference by unpaired t test model between two days from native PGT121 treated animals. C) Comparison between the above native control group and n-PGT121 treated group on PGT121 concentration in plasma on Day7 after infusion. The concentration of PGT121 was measured by ELISA in duplicates. Each symbol represents one individual rhesus macaque. ****: P values < 0.0001. D) Comparison between the above native control group and n-PGT121 treated group on PGT121 concentration in CSF on Day7 after infusion. The concentration of PGT121 in CSF was measured by ELISA in duplicates. Each symbol represents one individual rhesus macaque.
(DOCX)

S5 Fig The relation between vDNA in microglia and viral RNA copies in plasma and CSF of infant rhesus macaques with n-PGT121 treatment. A) vDNA in microglia is correlated with viral RNA in plasma in both Group I and Group II animals with n-PGT121 treatment. B) vDNA in microglia is not correlated with viral RNA in CSF in both Group I and Group II animals with n-PGT121 treatment.
(DOCX)

S1 Table Clinic histories of control infant rhesus macaques. (DOCX)

S2 Table Clinic histories of historical untreated and infant rhesus macaques treated with bNAbs and bNAbs+cART. (DOCX)

S3 Table SHIV_SF162P3_-associated viremia in the CNS of n-PGT121 treated infant rhesus macaques. (DOCX)
