# Supplementary material for: Improved delivery of broadly neutralizing antibodies by nanocapsules suppresses SHIV infection in the CNS of infant rhesus macaques
Source: PLoS Pathog. 2021 Jul 20;17(7):e1009738. doi: 10.1371/journal.ppat.1009738 (PMC8323878; doi:10.1371/journal.ppat.1009738)
Supplement: S1 Fig — The animals showing both positive RNA copies in plasma and vDNA are included in the figure. Each dot presents one animal, and all data are fitted by nonlinear log-log line regression. R2 and p value indicate the fitness of Pearson correlation. Dotted lines indicate limits of detection. (DOCX) [file ppat.1009738.s001.docx]

**S1 Fig** **Correlation between SHIV_SF162P3_ Gag RNA copies in plasma and vDNA copies in brain tissue.** The animals showing both positive RNA copies in plasma and vDNA are included in the figure. Each dot presents one animal, and all data are fitted by nonlinear log-log line regression. R^2^ and p value indicate the fitness of Pearson correlation. Dotted lines indicate limits of detection.
