# Supplementary material for: Improved delivery of broadly neutralizing antibodies by nanocapsules suppresses SHIV infection in the CNS of infant rhesus macaques
Source: PLoS Pathog. 2021 Jul 20;17(7):e1009738. doi: 10.1371/journal.ppat.1009738 (PMC8323878; doi:10.1371/journal.ppat.1009738)
Supplement: S2 Fig — The brain tissues of rhesus macaques were dissociated by papain, Dispase II, and DnaseI treatment, and followed by Percoll gradient sedimentation. Microglia (62%) were identified as CD45low CD11b+ cells, and macrophages (4%) were gated as CD45high CD11b high cells. (DOCX) [file ppat.1009738.s002.docx]

**Fig. S2** **Purification of isolated microglia from the brain tissue.** The brain tissues of rhesus macaques were dissociated by papain, Dispase II, and DnaseI treatment, and followed by Percoll gradient sedimentation. Microglia (62%) were identified as CD45^low^ CD11b+ cells, and macrophages (4%) were gated as CD45^high^ CD11b high cells.

**
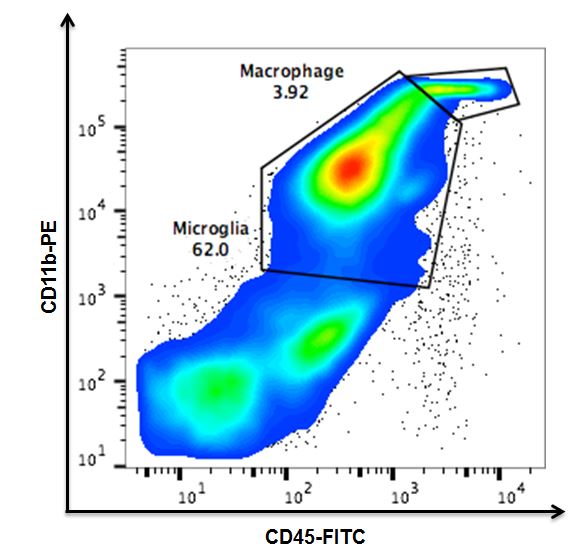
**
