# Supplementary material for: Improved delivery of broadly neutralizing antibodies by nanocapsules suppresses SHIV infection in the CNS of infant rhesus macaques
Source: PLoS Pathog. 2021 Jul 20;17(7):e1009738. doi: 10.1371/journal.ppat.1009738 (PMC8323878; doi:10.1371/journal.ppat.1009738)
Supplement: S3 Fig — A) The release kinetics of nanocapsules in PBS.10 μg of n-PGT121intact, n-PGT121, and n-PGT121hydrolysable were added into 1 ml PBS, respectively, and incubated at 37°C for 3 week. The concentration of released PGT121 was measured by ELISA. The concentration standard curve for ELISA was prepared with native PGT121. B) Nanocapsulation of PGT121 improves levels of its tissue penetration, including CNS, in rats. A single dose (10 mg/kg) of native and encapsulated PGT121 was administered in rats through tail-vein injection (n = 2). The concentrations of free PGT121 in plasma, CSF, and brain on Day 7. Brain tissues were collected from perfused animals, homogenized in PBS (1 mg tissue in 100 μl PBS), and tested by ELISA. C) Size of native PGT121 and n-PGT121 detected by dynamic light scattering (DLS). D) Representative transmission electron microscopy (TEM) image of n-PGT121. E) Evaluation of BBB leakage using evans blue (EB) in the brain tissues from mice treated with PBS, PGT121, and n-PGT121 one day post-injection. Brain tissues were harvested from mice after perfusion and homogenized for EB dye detection. Mice bearing brain tumors were used as positive control. EB dye fluorescence intensity was detected at 620/680 nm. Dye leakage was calculated from absorbance values to ng dye using a standard curve of EB in ethanol. (DOCX) [file ppat.1009738.s003.docx]

**Fig. S3** **Structure, morphology, and sustainable release of PGT121 nanocapsules (n-PGT121).** A) The release kinetics of nanocapsules in PBS.10 μg of n-PGT121_intact_, n-PGT121, and n-PGT121_hydrolysable_ were added into 1 ml PBS, respectively, and incubated at 37 °C for 3 week. The concentration of released PGT121 was measured by ELISA. The concentration standard curve for ELISA was prepared with native PGT121. B) Nanocapsulation of PGT121 improves levels of its tissue penetration, including CNS, in rats. A single dose (10 mg/kg) of native and encapsulated PGT121 was administered in rats through tail-vein injection (n = 2). The concentrations of free PGT121 in plasma, CSF, and brain on Day 7. Brain tissues were collected from perfused animals, homogenized in PBS (1 mg tissue in 100 μl PBS), and tested by ELISA. C) Size of native PGT121 and n-PGT121 detected by dynamic light scattering (DLS). D) Representative transmission electron microscopy (TEM) image of n-PGT121. E) Evaluation of BBB leakage using evans blue (EB) in the brain tissues from mice treated with PBS, PGT121, and n-PGT121 one day post-injection. Brain tissues were harvested from mice after perfusion and homogenized for EB dye detection. Mice bearing brain tumors were used as positive control. EB dye fluorescence intensity was detected at 620/680 nm. Dye leakage was calculated from absorbance values to ng dye using a standard curve of EB in ethanol.
