# Supplementary material for: Improved delivery of broadly neutralizing antibodies by nanocapsules suppresses SHIV infection in the CNS of infant rhesus macaques
Source: PLoS Pathog. 2021 Jul 20;17(7):e1009738. doi: 10.1371/journal.ppat.1009738 (PMC8323878; doi:10.1371/journal.ppat.1009738)
Supplement: S4 Fig — A) 10mg/kg of bNAb cocktail (5mg/kg PGT121 and 5mg/kg VRC07) was administered in rhesus macaques as the control group. Plasma and CSF were collected on Day1 and Day7 after infusion. The concentration of PGT121 in plasma and CSF was measured by ELISA in duplicates. Each symbol represents one individual rhesus macaque. B) The percentages of PGT121 CSF-concentration of plasma-concentration from infant rhesus macaques treated with native PGT121 on Day1 and Day7 after injection. There was no statistically significant difference by unpaired t test model between two days from native PGT121 treated animals. C) Comparison between the above native control group and n-PGT121 treated group on PGT121 concentration in plasma on Day7 after infusion. The concentration of PGT121 was measured by ELISA in duplicates. Each symbol represents one individual rhesus macaque. ****: P values < 0.0001. D) Comparison between the above native control group and n-PGT121 treated group on PGT121 concentration in CSF on Day7 after infusion. The concentration of PGT121 in CSF was measured by ELISA in duplicates. Each symbol represents one individual rhesus macaque. (DOCX) [file ppat.1009738.s004.docx]

**
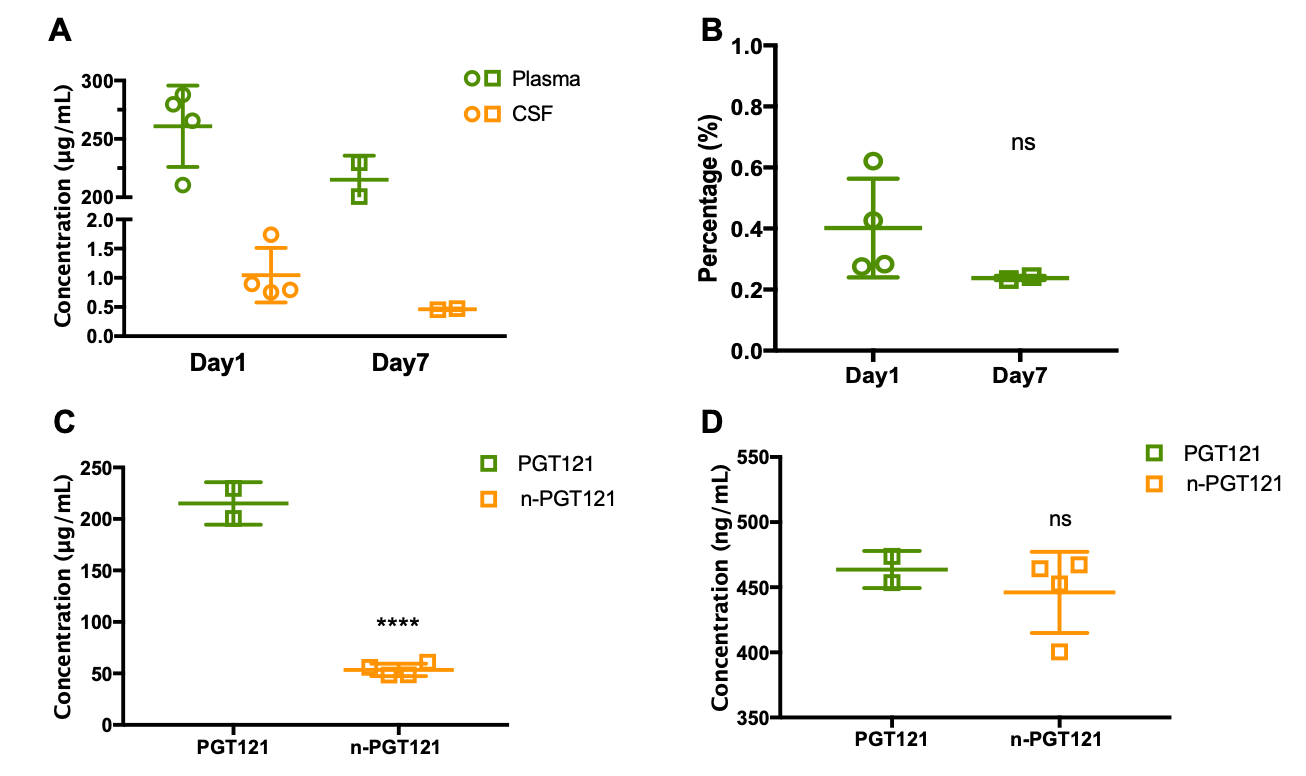
Fig. S4** **The nanocapsules improve PGT121 concentration in rhesus macaque CSF compared to native PGT121.** A) 10mg/kg of bNAb cocktail (5mg/kg PGT121 and 5mg/kg VRC07) was administered in rhesus macaques as the control group. Plasma and CSF were **collected** on Day1 and Day7 after infusion. The concentration of PGT121 in plasma and CSF was measured by ELISA in duplicates. Each symbol represents one individual rhesus macaque. B) The percentages of PGT121 CSF-concentration of plasma-concentration from infant rhesus macaques treated with native PGT121 on Day1 and Day7 after injection. There was no statistically significant difference by unpaired t test model between two days from native PGT121 treated animals. C) Comparison between the above native control group and n-PGT121 treated group on PGT121 concentration in plasma on Day7 after infusion. The concentration of PGT121 was measured by ELISA in duplicates. Each symbol represents one individual rhesus macaque. ****: P values < 0.0001. D) Comparison between the above native control group and n-PGT121 treated group on PGT121 concentration in CSF on Day7 after infusion. The concentration of PGT121 in CSF was measured by ELISA in duplicates. Each symbol represents one individual rhesus macaque.
