# Supplementary material for: Improved delivery of broadly neutralizing antibodies by nanocapsules suppresses SHIV infection in the CNS of infant rhesus macaques
Source: PLoS Pathog. 2021 Jul 20;17(7):e1009738. doi: 10.1371/journal.ppat.1009738 (PMC8323878; doi:10.1371/journal.ppat.1009738)
Supplement: S5 Fig — A) vDNA in microglia is correlated with viral RNA in plasma in both Group I and Group II animals with n-PGT121 treatment. B) vDNA in microglia is not correlated with viral RNA in CSF in both Group I and Group II animals with n-PGT121 treatment. (DOCX) [file ppat.1009738.s005.docx]

**
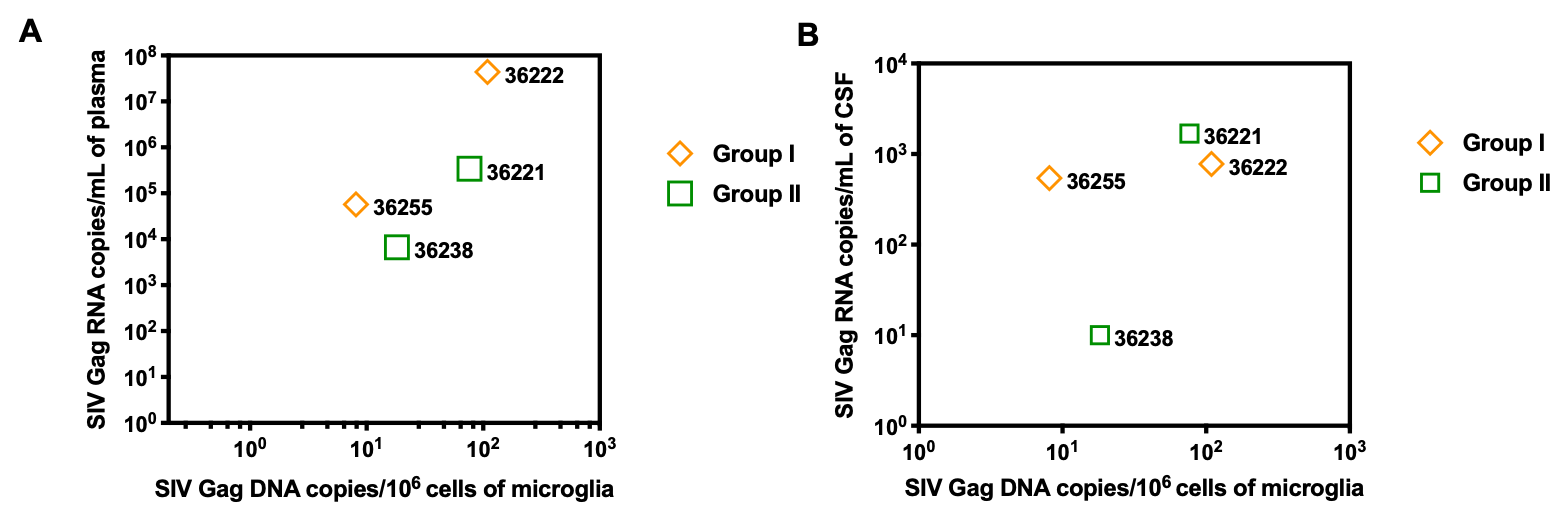
Fig. S5 The relation between vDNA in microglia and viral RNA copies in plasma and CSF of infant rhesus macaques with n-PGT121 treatment.** A) vDNA in microglia is correlated with viral RNA in plasma in both Group I and Group II animals with n-PGT121 treatment. B) vDNA in microglia is not correlated with viral RNA in CSF in both Group I and Group II animals with n-PGT121 treatment.
