# Supplementary material for: Improved delivery of broadly neutralizing antibodies by nanocapsules suppresses SHIV infection in the CNS of infant rhesus macaques
Source: PLoS Pathog. 2021 Jul 20;17(7):e1009738. doi: 10.1371/journal.ppat.1009738 (PMC8323878; doi:10.1371/journal.ppat.1009738)
Supplement: S1 Table — (DOCX) [file ppat.1009738.s006.docx]

**Table S1 Clinic histories of control infant rhesus macaques**

| **Animal Number** | 33172 | 33186 | 34932 | 34947 | 35364 | 35355 | 35378 | 36243 | 36206 | 36207 | 37223 |
| --- | --- | --- | --- | --- | --- | --- | --- | --- | --- | --- | --- |
| **Gender** | M | M | F | F | M | M | M | M | F | M | M |
| **Virus Dose (mL)** | 0.5 | 0.5 | 0.5 | 0.5 | 0.5 | 0.5 | 0.5 | 1 | 2 | 2 | 2 |
| **Time of necropsy**  **(Post-infection)** | WK2 | WK2 | WK1 | WK1 | WK22 | WK21 | WK24 | WK15 | WK2 | WK2 | WK10 |
| **RNA copies/mL of plasma** | 5.7×10^7^ | 4.6×10^7^ | 2.9×10^6^ | 2.0×10^7^ | 1.3×10^7^ | 3.1×10^7^ | 1.0×10^2^ | 2.1×10^6^ | 8.6×10^4^ | 3.4×10^5^ | 1.0×10^8^ |
| **RNA copies/mL of CSF** | 4.1×10^4^ | 3.3×10^4^ | 8.5×10^1^ | 1.2×10^4^ | 3.4×10^3^ | 2.2×10^3^ | 4.4×10^1^ | 3.4×10^3^ | 3.4×10^2^ | 7.3×10^2^ | 6.5×10^4^ |
| **DNA copies/10^6^ brain cells** | 3.2×10^1^ | 4.1×10^1^ | 4.6×10^0^ | 6.8×10^0^ | x | x | x | 0.8×10^0^ | x | x | x |
