# Supplementary material for: Improved delivery of broadly neutralizing antibodies by nanocapsules suppresses SHIV infection in the CNS of infant rhesus macaques
Source: PLoS Pathog. 2021 Jul 20;17(7):e1009738. doi: 10.1371/journal.ppat.1009738 (PMC8323878; doi:10.1371/journal.ppat.1009738)
Supplement: S2 Table — (DOCX) [file ppat.1009738.s007.docx]

**Table S2 Clinic histories of historical untreated and infant rhesus macaques treated with bNAbs and bNAbs+cART**

| **Group** | Untreated | Treated | | | |
| --- | --- | --- | --- | --- | --- |
| **Infection Dose** | 2mL | 2mL | 2mL | 0.5mL | 2mL |
| **Treatment** | / | 10mg/kg bNAb cocktail  (D2-D7) | cART (D2-D22) +10mg/kg bNAb (D2-D7) | 10mg/kg bNAb(D2-D7) | 10mg/kg single bNAb (D2-D7) |
| **Animal Numbers** | 11 | 3 | 3 | 5 | 3 |
