# Supplementary material for: Improved delivery of broadly neutralizing antibodies by nanocapsules suppresses SHIV infection in the CNS of infant rhesus macaques
Source: PLoS Pathog. 2021 Jul 20;17(7):e1009738. doi: 10.1371/journal.ppat.1009738 (PMC8323878; doi:10.1371/journal.ppat.1009738)
Supplement: S3 Table — (DOCX) [file ppat.1009738.s008.docx]

**Table S3 SHIV_SF162P3_-associated viremia in the CNS of n-PGT121 treated infant rhesus macaques**

| **Group** | Group I  Day2 and WK11 | | | | Group II  WK11 | |
| --- | --- | --- | --- | --- | --- | --- |
| **Animal ID** | 36208 | 36220 | 36222 | 36255 | 36221 | 36238 |
| **Tissues** | SIV_gag_ DNA copies/μg of tissue DNA | | | | | |
| **CSF^#^** | 0 | 0 | 779.87 | 543.96 | 1683.07 | 0 |
| **Brain** | 0 | 0 | UD* | 0.51 | UD* | 0.07 |
| **Microglia** | 0 | 0 | 16.74 | 1.25 | 11.77 | 2.8 |

UD*: Undetermined

CSF^#^: the unit of VL in CSF is SIVgag RNA copies/mL
